# Homoeologous evolution of the allotetraploid genome of *Poa annua* L.

**DOI:** 10.1186/s12864-023-09456-5

**Published:** 2023-06-26

**Authors:** Christopher W. Benson, Matthew R. Sheltra, Peter J. Maughan, Eric N. Jellen, Matthew D. Robbins, B. Shaun Bushman, Eric L. Patterson, Nathan D. Hall, David R. Huff

**Affiliations:** 1 Department of Plant Science, Pennsylvania State University, University Park, PA USA; 2 Intercollegiate Graduate Degree Program in Plant Biology, Pennsylvania State University, University Park, PA USA; 3 Department of Plant and Wildlife Sciences, Brigham Young University, Logan, UT USA; 4 USDA ARS, Forage and Range Research, Logan, UT USA; 5 Department of Plant, Soil, and Microbial Sciences, Michigan State University, East Lansing, MI USA

**Keywords:** Chromosomal rearrangements, Transposable elements (TEs), Retrotransposons, Phenotypic plasticity, Allopolyploid, Whole-genome duplication (WGD), Genome evolution, Genome sequencing, Weed, Turfgrass

## Abstract

**Background:**

*Poa annua* (annual bluegrass) is an allotetraploid turfgrass, an agronomically significant weed, and one of the most widely dispersed plant species on earth. Here, we report the chromosome-scale genome assemblies of *P. annua’s* diploid progenitors, *P. infirma* and *P. supina,* and use multi-omic analyses spanning all three species to better understand *P. annua’s* evolutionary novelty.

**Results:**

We find that the diploids diverged from their common ancestor 5.5 – 6.3 million years ago and hybridized to form *P. annua* ≤ 50,000 years ago. The diploid genomes are similar in chromosome structure and most notably distinguished by the divergent evolutionary histories of their transposable elements, leading to a 1.7 × difference in genome size. In allotetraploid *P. annua,* we find biased movement of retrotransposons from the larger (A) subgenome to the smaller (B) subgenome. We show that *P. annua’s* B subgenome is preferentially accumulating genes and that its genes are more highly expressed. Whole-genome resequencing of several additional *P. annua* accessions revealed large-scale chromosomal rearrangements characterized by extensive TE-downsizing and evidence to support the Genome Balance Hypothesis.

**Conclusions:**

The divergent evolutions of the diploid progenitors played a central role in conferring onto *P. annua* its remarkable phenotypic plasticity. We find that plant genes (guided by selection and drift) and transposable elements (mostly guided by host immunity) each respond to polyploidy in unique ways and that *P. annua* uses whole-genome duplication to purge highly parasitized heterochromatic sequences. The findings and genomic resources presented here will enable the development of homoeolog-specific markers for accelerated weed science and turfgrass breeding*.*

**Supplementary Information:**

The online version contains supplementary material available at 10.1186/s12864-023-09456-5.

## Background

Polyploidy, or whole-genome duplication (WGD), is a repeated phenomenon in the evolution of plants and frequently associated with the emergence of novel traits, elevated stress tolerance, and niche expansion. In addition to the abundance of young and recently formed polyploids, it is now evident that all angiosperms have remnants of ancient WGD [1, 2]. Polyploidy can influence cellular processes, including transposable element (TE) activity, gene expression changes, epigenetic modifications, and chromosomal restructuring [3, 4]. Allopolyploidy is a type of WGD that arises when two or more distinct species hybridize through an interspecific cross. The merged genomes of an allopolyploid are referred to as subgenomes and are ancestrally related but have separate evolutionary histories. Ancestrally related chromosomes between the subgenomes are called homoeologs and share similar structure and gene orientation. Allopolyploids predominantly use bivalent chromosome pairing during meiosis, but when bivalence fails, homoeologs can recombine and homoeologous exchanges (HEs) can occur [5, 6]. After many generations (or possibly only a few) [7], most allopolyploids eventually establish a ‘dominant’ subgenome, with higher expression of homoeologs and fewer lost genes (less fractionated) as the species returns to a diploid-like state (diploidization) [8–12]. Interestingly, recent work suggests that certain features of the parental genomes might help predispose subgenomes for dominance after allopolyploidy [13, 14]. For example, TE density may be a useful predictor of subgenome dominance because silencing TEs can involve methylation spillover to nearby genes, which can reduce their expression relative to the less TE dense homoeolog [10, 15]. Studies focused on WGD are challenging due to the high sequence similarity between homoeologs but will be instrumental to better understanding the *cis–trans* regulatory relationships that govern phenotypic plasticity in allopolyploid crops.

**Fig. 1:**
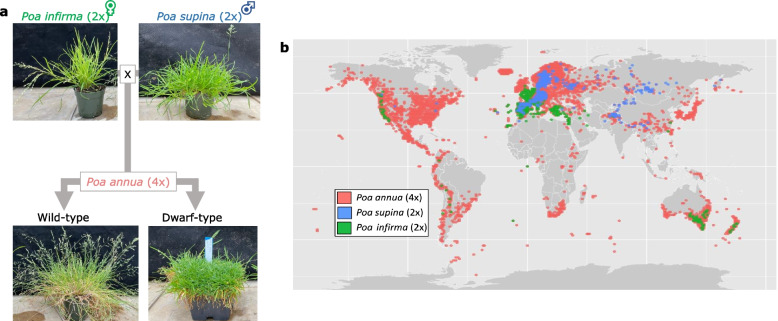
The evolutionary origin of allotetraploid *Poa annua*. **a** Images of parental diploids, *P. infirma* and *P. supina*, and derived allotetraploid, *P. annua*. Two biotypes of *P. annua* are shown; a wild-type plant with annual lifespan and upright growth and a dwarf-type plant with perennial lifespan and prostrate growth. Grey arrows indicate the parental relationship between species. **b** The present-day geographic ranges of diploid and allotetraploid *Poa* species shows transgressive versatility and niche expansion of *P. annua*. Coordinate data downloaded from the Global Biodiversity Information Facility on 9/19/2021 with Antarctic additions according to Chwedorzewska et al*.,* [22]

One of the most ubiquitous allopolyploids on earth is the grass species, *Poa annua* L. (2*n* = 4*x* = 28). *Poa annua* is an allotetraploid that originated from an interspecific cross between diploid species, *Poa infirma* Kunth and *Poa supina* Schrader (Fig. 1a) [16–19]. The parental diploids of *P. annua* are restricted to their niches where *P. infirma* thrives in arid Mediterranean climates and *P. supina* prefers the boreal and alpine regions of central Europe. In contrast to its progenitors, *P. annua* has remarkable phenotypic variability that has allowed it to establish seeding populations on all seven continents and 96% of cities around the world (Fig. 1b) [20–22]. It is a problematic weed in urban, agricultural, and turfgrass ecosystems, partially due to its evolved resistance to more than 10 different herbicide modes of action [23]. Despite its unfavorable reputation, *P. annua* has developed an agronomic niche on golf course putting greens where it often invades and outcompetes turfgrass species that were bred to thrive under the intensive management conditions of 2-3 mm mowing height [24]. Some golf course superintendents come to view *P. annua* as an elite putting surface and allow it to slowly envelope the entire putting green. In fact, seven of the top ten golf courses in the United States utilize *P. annua* putting greens (top100golfcourses.com).

The parental diploids of *P. annua* can hybridize at low frequencies (0.20%) and the offspring are amphihaploid (polyhaploid; i.e., plants that contain a single set of unpaired chromosomes for each subgenome) [25]. Amphihaploid plants (2*n* = 14) are sterile at first but have been observed to spontaneously transition to fertile allotetraploids (2*n* = 28) [26], suggesting that *P. annua’s* path to polyploidy may have involved mitotic (somatic doubling) rather than meiotic error (unreduced gametes). Interestingly, amphihaploids are frequently found on golf course putting greens [27], suggesting that polyploid *P. annua* can return to amphihaploidy [28, 29] in certain environmental conditions and may oscillate between the two cytotypes. Here, we leverage the genomes of the diploid progenitors to accurately assign *P. annua* homoeologs to their appropriate parental origin. Using this methodology, we unravel *P. annua’s* polyploid evolutionary history with the goal to better understand its phenotypic plasticity and provide a valuable genetic resource for turfgrass breeders and weed scientists.

## Results

### Genome assembly and annotation

The *P. infirma* (2*n* = 2*x* = 14) and *P. supina* (2*n* = 2*x* = 14) genomes each assembled into seven pseudomolecules that represented 96% of the estimated genome sizes by k-mer analysis and contained > 97% of the 1,614-core conserved orthologs in the Embryophyta OrthoDB (v10), supporting high-quality chromosome-level genome assemblies for both species (see methods; Supplementary Table 1; Supplementary Fig. 1). The chromosome-level assemblies represent the collapsed haploid (unphased) genomes for each species (*n* = 7). Chromosomes were named according to a pre-established nomenclature presented by Robbins et al*.* [30], where *P. infirma* contributes the ‘A’ subgenome to *P. annua* and *P. supina* contributes the ‘B’ subgenome. A prefix designates the species of origin, such that *P. infirma* chromosomes are ‘PiA’, *P. supina’s* are ‘PsB’, and *P. annua’s* are either ‘PaA’ or ‘PaB’ (Supplementary Fig. 2a).

Repetitive DNA and TEs were annotated using custom built repeat libraries and included class I retrotransposons as well as class II DNA transposons. Genes were predicted using the BRAKER2 pipeline on the repeat-masked genome assemblies [31]. Full-length Iso-Seq transcripts from each species was incorporated with protein evidence from *Arabidopsis* and related grasses for ab initio gene prediction. In addition, we identified 14,743 long noncoding RNAs (lncRNAs) in the *P. infirma* genome and 13,963 in the *P. supina* genome. *Poa annua* contained approximately the additive number of lncRNAs as its diploid parents with fewer lncRNAs in the A (infirma) subgenome (14,394) and more lncRNAs in the B (supina) subgenome (15,057).

### Genome characteristics and synteny

**Fig. 2:**
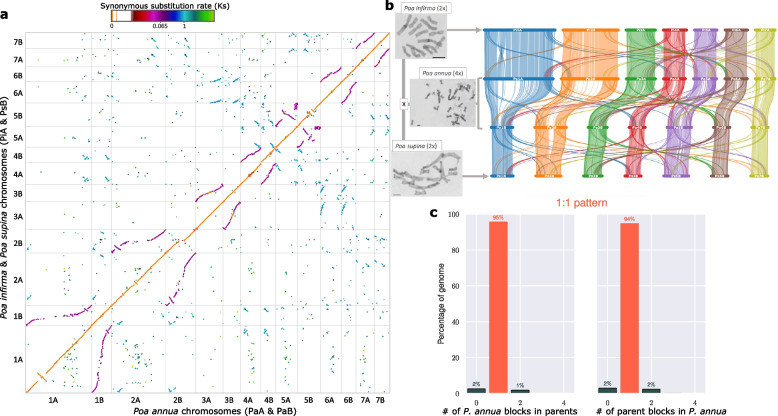
The comparative colinear relationship of three *Poa* genomes*.* **a** Macrosyntenic comparison of the *P. annua* genome (PaA & PaB; x-axis) to the combined *P. infirma* (PiA) and *P. supina* (PsB) genomes (y-axis). PiA to PaA and PsB to PaB comparisons are orange. Breaks in the contiguity of the orange line illustrate recent structural modifications occurring after the hybridization of the tetraploid (post-polyploidy). PiA to PaB and PsB to PaA comparisons are purple and illustrate structural modifications that occurred after the parental diploid species diverged from their common ancestor. The ‘S’ curve in some syntenic comparisons illustrates differences in chromosome size. Colors denote synonymous substitution rate (Ks). The Ks values in the scale bar indicates three important events: polyploid hybridization (Ks = 0), speciation of the parents (Ks = 0.065), and the rho (ρ) WGD event (Ks = 1). **b** The photomicrograph and syntenic ribbon plot depict the relative chromosome size, structure, and collinear relationship between the genomes of the *P. infirma, P. supina,* and allotetraploid *P. annua.* Scale bar in the bottom corner of the photomicrograph indicates the relative chromosome sizes. **c** The ratio of syntenic depth between genes of the diploid parents and genes of *P. annua* indicate a 1:1 relationship

The *P. infirma* genome is 1,125 Mb in length, which makes it 489 Mb (1.77 ×) larger than the *P. supina* genome (636 Mb), despite being closely related species and sister taxa within the section *Micrantherae* (syn. *Ochlopoa*). Most (76%) of the excess in genome size is due to orthologous chromosomes 1 and 2 being a combined 374 Mb larger in the *P. infirma* genome (Fig. 2ab; Supplementary Fig. 3). The subgenomes of *P. annua* are similar in composition to the genomes of the diploid progenitors, with the A subgenome (1,116 Mb) being 1% shorter than the *P. infirma* genome and the B subgenome (662 Mb) being 4% larger than the *P. supina* genome (Supplementary Fig. 2c)*.* At the gene level, the A (infirma) subgenome had 6% fewer genes than *P. infirma* (37,123 and 39,420, respectively), and the B (supina) subgenome had 4% more genes than *P. supina* (39,536 and 37,935 respectively). Overall, the *P. annua* reference genome is 99% of the length of its progenitor genomes and contains 99% of its parental genes, most of which (95%) are represented as colinear syntenic blocks (Fig. 2c; Supplementary Fig. 2c; Supplementary Fig. 4).

*Poa infirma* and *P. supina* chromosomes were 81% and 65% repetitive, respectively. These percentages amount to 489 Mb (1.77 ×) more repetitive DNA in *P. infirma* than *P. supina,* suggesting that TEs have played an outsized role in the disparate genome sizes between the two diploids, particularly on orthologous chromosomes 1 and 2. The majority of annotated repetitive sequences were classified as Gypsy and Copia long terminal repeat (LTR) retrotransposons (598 Mb (53%) of the *P. infirma* genome and 241 Mb (38%) of the *P. supina* genome). The sequence length of the non-repetitive portions in each diploid is very similar, totaling 211 Mb in the *P. infirma* genome and 225 Mb in the *P. supina* genome. The subgenomes of *P. annua* have slightly less repetitive DNA than their corresponding diploid progenitor genomes, with 7% less repetitive DNA in the A (infirma) subgenome and 2% less in the B (supina) subgenome.

### Nucleotide divergence, molecular dating, and bursts of LTRs

Genomic similarity can be assessed at the nucleotide level using measures of average nucleotide identity (ANI) and is a useful indicator of genetic divergence between sequence alignments. The ANI between *P. infirma* (A) and *P. supina* (B) orthologous chromosomes is 95%. The ANI when comparing *P. annua* chromosomes to their corresponding parental sequences was 98% (i.e., PaA to PiA alignments and PaB to PsB; Supplementary Fig. 2b). To estimate divergence and hybridization times, we calculated the synonymous substitutions rate (Ks) between homologous and homoeologous gene pairs. Gene pairs between *P. infirma* (A) and *P. supina* (B) have a peak Ks = 0.065 and was used to estimate the date that the two species diverged from their common ancestor. Ks between *P. annua’s* A subgenome and *P. infirma* (and also *P. annua’s* B subgenome and *P. supina*) was very close to zero and used to estimate the date that the two progenitor diploids hybridized to form *P. annua*. With a Poaceae mutational rate of 5.76174 × 10^–9^ substitutions per synonymous site per year [32], our Ks values suggest that the diploids diverged from their common ancestor 5.5 – 6.3 million years ago (Mya) and hybridized to form polyploid *P. annua* 0 – 600,000 years ago (Supplementary Fig. 5). The most recent of the ancestral WGD events in the Poaceae is rho (ρ) and pre-dates the divergence of the BOP (C3) and PACMAD (C4) grasses [33]. Syntenic gene pairs from rho have a Ks = 1 in our *Poa* species and corresponds to a date of 87 Mya, which largely overlaps with the reported rho WGD date of 85–97 Mya and helps to corroborate our methodology (Supplementary Fig. 6) [34]. Furthermore, our estimated date of diploid divergence is in agreement with a recent analysis based on plastid markers [35].

**Fig. 3:**
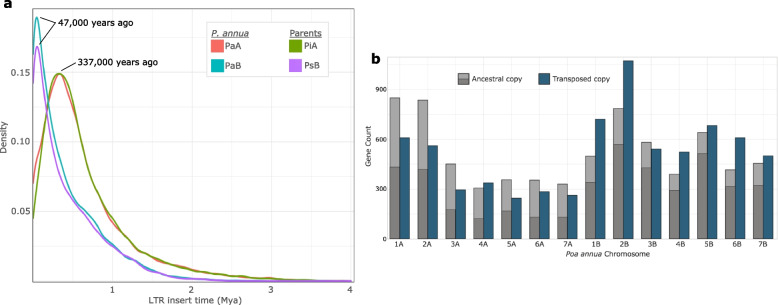
Retrotransposon mobility in *Pa annua.* **a** Dating the insertion times of LTRs shows varying bursts of mobility in the subgenomes of *P. annua* (PaA & PaB) and its diploid progenitors, *P. infirma* (PiA) and *P. supina* (PsB). **b** Transposed gene duplications that mobilized post-polyploidy (0 – 50,000 years ago) show biased movement from the A subgenome (left) to the B subgenome (right). Light grey are ancestral copies that translocated to the opposite subgenome (inter-subgenome), while dark grey are ancestral copies that translocated and stayed within their parental subgenome (intra-subgenome). Blue is the location of the novel (transposed) copy. Transposed gene duplications are heavily enriched for LTR-associated activity

To further evaluate the date of hybridization and explore the 1.7-fold difference in genome size between A and B, we examined the mutation rates between pairs of LTRs. LTRs multiply by escaping host silencing and ‘burst’ into activity for a short time before being re-silenced [36, 37]. Repeats of an LTR are identical when inserted, owing to their copy-and-paste mode of transposition [38]. Mutations between an ancestral LTR and its transposed derivative are a reflection of its evolutionary divergence. Our analysis suggests that the A genome experienced a burst in proliferation of LTRs that climaxed ~ 340,000 years ago, while bursts of LTRs in the B genome occurred more recently, with peak rate of transposition dating back to ~ 50,000 years ago (Fig. 3a). Because the density of LTR insertion times in *P. infirma* and *P. supina* closely mirror that of *P. annua’s* A and B subgenomes, it is likely that those bursts occurred during the speciation of the diploids and prior to the hybridization event that formed *P. annua.* Thus, we suggest a narrower timeframe for *P. annua* hybridization at 0 – 50,000 years ago*.* We expect that the 489 Mb difference in TE content and genome size between *P. infirma* and *P. supina* is greatly impacted by the two species varying abilities to silence retrotransposons.

### Single-gene duplications and retrotransposon activity

In addition to the WGD that formed *P. annua*, smaller scale duplications can also accompany polyploidy and are collectively referred to as single-gene duplications [39]. We identified 2,008 tandemly duplicated and 1,815 proximally duplicated genes in the *P. infirma* genome*.* These numbers are similar to *P. supina* with 1,940 tandem and 1,914 proximal duplications. As compared to its progenitor genomes*,* allotetraploid *P. annua* has slightly fewer single-gene duplications in the A (infirma) subgenome (1,806 tandem and 1,736 proximal duplicated genes), and slightly more in the B (supina) subgenome (1,999 tandem and 2,160 proximal). Transposed duplications are another type of single-gene duplication and are thought to occur extensively after polyploidy [40–42]. We used the progenitor *P. infirma* and *P. supina* genomes as outgroups to identify pairs of transposed genes that were mobilized after the diploids hybridized to form *P. annua* (post-polyploidy). We found 63% more transposed duplications in *P. annua’s* B subgenome than in *P. annua’s* A subgenome (5,917 and 3,438 transposed genes, respectively). This result is similar to the pattern observed with proximal and tandem duplications and may point to a post-polyploidy expansion of the B subgenome and contraction of the A subgenome within *P. annua*.

Interestingly, 74% of transposed duplications in the B subgenome remained within B, while 46% of A duplications remained within the A subgenome, suggesting that inter-subgenomic duplications preferentially move from the A (infirma) subgenome and integrate into B (supina; Fig. 3b; χ^2^ test, *P* < 0.0001). Inter-subgenomic transposed duplications are enriched for functions associated with Gypsy and Copia-type LTRs, suggesting that they are heavily involved with retrotransposon activity. Taken together with our molecular dating of LTRs, we expect that the observed bias in inter-subgenome transpositions is a reflection of the two subgenomes uneven abilities to inhibit retrotransposons and is a continuation of the TE momentum that was established during the independent evolutions of the diploids. The observed bias in inter-subgenome transpositions may point to a *trans* relationship, where retrotransposons ‘diffuse’ from the subgenome with higher TE content to the subgenome with lower TE content.

### Homoeologous exchanges

**Fig. 4:**
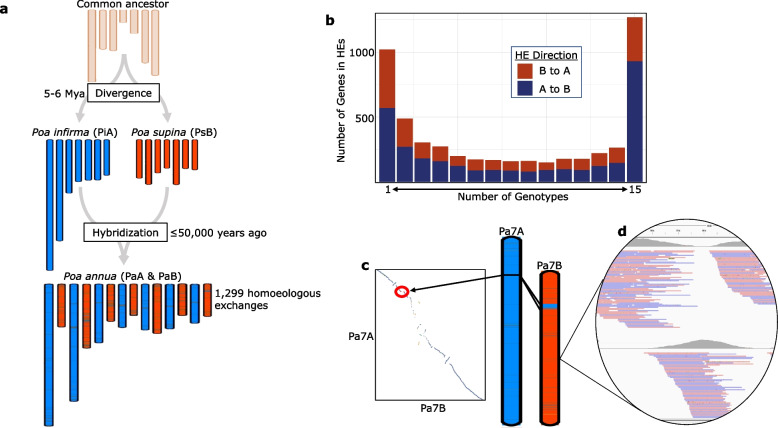
Homoeologous exchanges in *Poa annua.* **a** A karyotypic view of *P. annua’s* allotetraploid evolution illustrated by the haplotig-level genome assemblies. Chromosome lengths are scaled according to their relative size. Karyotype of the common ancestor is unknown, and a theoretical karyotype is depicted. **b** A bimodal distribution of genes within HEs across 15 re-sequenced *P. annua* genotypes shows biased reshuffling favoring gene movement to the B subgenome. On the left are genes found in rare HEs that occur in one or a few genotypes. On the right, genes commonly found within HEs occurring in most or all genotypes. **c** A graphical depiction of the largest HE in the *P. annua* genome. Circled in the syntenic dotplot, the disjunction highlights that the 2.2 Mb HE is an unbalanced Pa7A to Pa7B translocation. **d** An IGV alignment window shows a HE in the *P. annua* genome. *Poa annua* reads are tagged by their parental origin and mapped to the *P. annua* reference genome (see methods). Reads with *P. supina* origin are shown in the top half of the image, while reads with *P. infirma* origin are on the bottom half. Because this is a B subgenome chromosome (Pa7B), regions with *P. infirma* origin are putative HEs

Crossing over between ancestrally related chromosomes is a common occurrence in newly formed allopolyploids and are referred to as HEs [43, 44]. We assessed HEs in *P. annua* (i.e., A segments in the B subgenome and B segments in the A subgenome) using the parental sequences as a guide to assign *P. annua* reads as either being derived from *P. infirma* or *P. supina*. We detected 1,299 HEs in the *P. annua* genome (Fig. 4; 657 A segments in the B subgenome and 642 B segments in the A subgenome). Almost 2% of *P. annua’s* gene annotations are within HEs. Of those, 68% are A to B, suggesting that there may be an asymmetric exchange of genic sequences between the two subgenomes (823 A genes in the B subgenome vs 385 B genes in the A subgenome; χ^2^ test, *P* < 0.0001). The average length of an HE was 16 kb for A to B subgenome HEs and 13 kb for B to A subgenome HEs. Interestingly, 1.6% of the B subgenome consists of A sequences (10.4 Mb), while 0.7% of the A subgenome is B sequences (8.3 Mb). A to B HEs were most enriched for genes involved in gibberellin 3-beta-dioxygenase activity, while B to A HEs were enriched in genes involved in telomere maintenance. The largest HE is a 2.2 Mb Pa7A to Pa7B exchange containing 103 genes (Fig. 4c). Three of *P. annua’s* 26 annotated histone H3-K4 methylation genes reside in this 2.2 Mb HE. The differences in HEs between subgenomes points to a visible but tenuous bias accumulation of genes in the B subgenome.

### Fractionation bias

Gene loss (fractionation) occurs via intrachromosomal recombination resulting in short deletions and is a typical behavior of ancient allopolyploids [45]. We compared the A and B subgenomes of *P. annua* to the A and B genomes of its progenitors and identified consistent gene retention (97%) across all chromosomes, likely reflecting the recent timescale of the *P. annua* WGD event (Supplementary Fig. 7). Although this result seems to clash with our observations at the single-gene and HE levels, it is important to note the distinction between these methodologies. The fractionation analysis used here [46] calculates the number of genes retained in *P. annua* with respect to the syntenic sequences in the progenitor genomes. Consequently, single-gene duplications would only impact our fractionation analysis if they had duplicated in the progenitor genome but not in *P. annua.* The impact of HEs on our fractionation analysis is relatively small, since there are only 1,208 genes within HEs and most (~ 61%) have an ancestrally syntenic ortholog in the homoeologous subgenome and therefore would not impact fractionation values.

### Homoeolog expression bias and polyploid plasticity

*P. annua* is typically described as having two distinct biotypes; plants with wild-type morphology and plants with dwarf-type morphology (Fig. 1a; sometimes referred to as annual- and perennial-types, respectively) [47]. Plants with wild-type habit resemble *P. infirma,* while the dwarf-types more closely resemble *P. supina.* Genetic factors contribute to *P. annua’s* morphology, but broad phenotypic plasticity has also been reported where environmental stressors such as animal disturbance, intense wind, soil properties, temperature, elevation, and even golf course-style management can influence plants to preferentially favor one biotype over the other [48]. The two contrasting morphologies likely play an important role in *P. annua’s* ability to infiltrate and persist across a spectrum of climactic conditions [49].

**Fig. 5:**
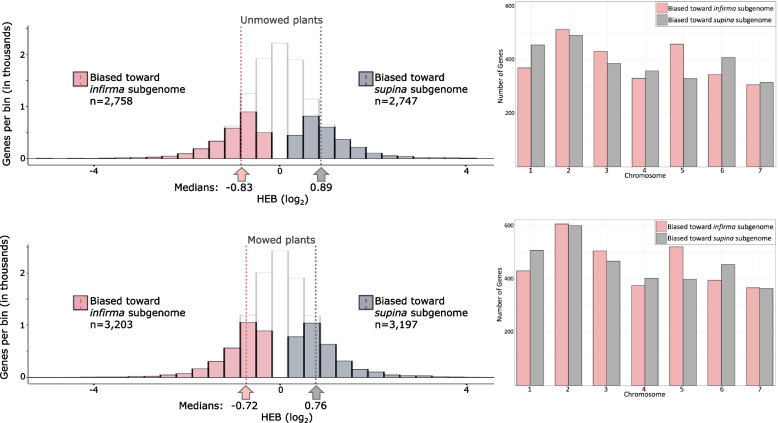
Homoeolog expression bias tests the polyploid plasticity hypothesis under golf course-style mowing stress*.* Clonally propagated plants were exposed to mowing stress (bottom), or not exposed to mowing stress (top). In the histograms, solid bars are homoeologous gene pairs with a FDR ≤ 0.05. Open bars include all testable gene pairs. Pink are significantly biased toward the A (infirma) subgenome and grey are significantly biased toward the B (supina) subgenome. The bar plots adjacent to the histograms show differentially expressed genes across all seven homoeologous pairs of chromosomes

Shimizu-Inatsugi et al*.* [50] introduced the Polyploid Plasticity Hypothesis stating that an allopolyploid species might differentially utilize the expression profiles of its progenitor genomes depending on the environment. With agronomic and turfgrass breeding in mind, we aimed to test the hypothesis that *P. annua* might preferentially express genes from the B (supina) subgenome when exposed to mowing stress and the A (infirma) subgenome when allowed to grow in the absence of mowing stress (unmowed). We vegetatively propagated dwarf- and wild-type *P. annua* plants and subjected one clone to mowing stress for three months, while leaving the other clone unmowed for three months. We observed no correlation in the expression profiles between biotypes (dwarf or wild) across our biological replicates (Supplementary Fig. 8; Supplementary Fig. 9), indicating that dwarf-types and wild-types exhibit similar transcriptional behavior under both mowed and unmowed conditions. After removing biotypes as a variable, we identified 5,505 and 6,400 differentially expressed pairs of homoeologs in our unmowed and mowed comparisons, respectively. We found that both mowed and unmowed plants showed a homoeolog expression bias favoring the B subgenome (Wilcoxon test: *p* = 0.001 and *p* = 0.0008, respectively), indicating that *P. annua* preferentially utilizes B (supina) genes regardless of mowing stress (Fig. 5). Although *P. annua’s* B subgenome expression bias is statistically significant in both treatment comparisons, the bias is not as evident as reported in other neo-allopolyploids [7, 51–53], likely reflecting the recent timescale of the hybridization but perhaps also pointing to a more equitable relationship between *P. annua’s* subgenomes where primary metabolic function is partitioned across pairs of homoeologs (Supplementary Fig. 10). Only chromosomes one, four, and six showed consistent expression bias toward B homoeologs, suggesting that these three chromosomes contribute disproportionally to homoeolog expression bias at the whole-genome level (Fig. 5). Thus, we conclude that counter to the polyploid plasticity hypothesis, *P. annua* utilizes genes from both subgenomes with modest homoeolog expression bias favoring B (supina) genes irrespective of mowing treatments.

In addition to homoeolog expression analysis, we also used our transcriptional data to compare gene expression between pairs of recently transposed gene duplications that were identified during our analysis of single-gene duplications. We identified, 973 pairs of transposed genes as being differentially regulated between their novel and ancestral copies. Of those, 847 (87%) were upregulated in the novel copy and most were A to B transpositions.

### Whole-genome resequencing and large-scale chromosomal modifications

Homoeologous exchanges and bursts of activity in transposable elements contribute to genomic instability in polyploids but do not provide a satisfying explanation for the reported 80% variation in DNA content between *P. annua* genotypes [54, 55]. To explore intraspecific variation in *P. annua* at the whole-chromosome and DNA sequence level, we re-sequenced 13 geographically distinct accessions and two additional elite breeding lines. Together, the 15 samples represent nine countries and four continents (Supplementary Fig. 11). The Illumina reads were aligned to the *P. annua* reference genome with a depth of coverage ranging between 13–26 ×. More than 99% of all reads mapped to the *P. annua* reference genome. SNP density across a 1 Mb sliding window showed large variability in sequence divergence within subgenomes, suggesting that there may have been multiple hybrid origins (Supplementary Fig. 12). Of the 76,541 gene annotations in the reference genome, we found that 7,808 were absent (dispensable) from at least one of the 15 samples, leaving 68,733 ‘core’ genes approximately evenly split between subgenomes (Supplementary Fig. 13; 52% of core genes were from the B subgenome). Dispensable genes were enriched for function in RNA-mediated transposon integration, suggesting that retrotransposons are actively proliferating in the species in a genotype-specific manner. In addition to core and dispensable genes, we used the diploid genomes to identify HEs and determine the parental origin for *P. annua* homoeologs across all 15 samples. There were 5,217 genes within HEs in at least one sample. A to B HEs were enriched for functions associated with primary metabolism, while B to A HEs were enriched for functions associated with telomere maintenance. Most (60%) genes within HEs were transferred from the A to the B subgenome, continuing to point toward a biased accumulation of genes in B (supina) homoeologs (Fig. 4b; χ^2^ test, *P* < 0.0001).

**Fig. 6:**
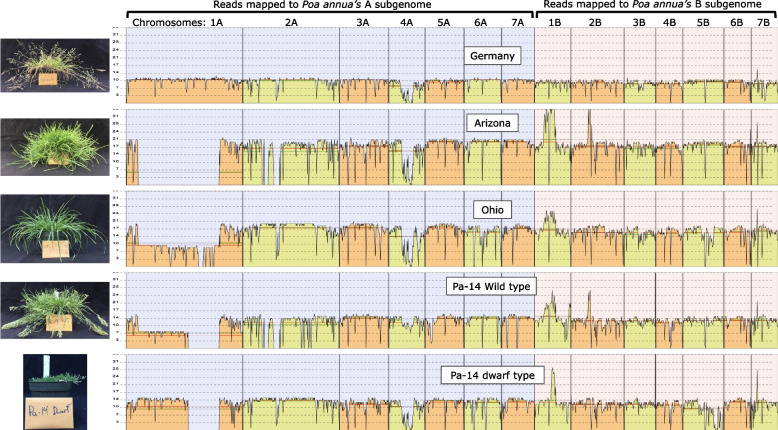
Depth of coverage plotted along the *Poa annua* reference genome shows large-scale variation in chromosome structure. Regions of reduced coverage indicate deletions relative to the reference genome, while regions with elevated coverage show duplications. The *P. annua* reference genome is unphased (haplotypes are collapsed), so regions with 1/2 × coverage indicate chromosomes with haplotype-specific duplications and deletions (heterozygotes). Samples ‘Germany’ and ‘Arizona’ were imaged 125 days after germination. ‘Ohio’ was imaged 56 days after germination. The two breeding lines (‘Pa-14’ dwarf and wild) were collected from a field experiment and allowed to grow unmowed for 56 days prior to imaging. Mean and median map coverage are indicated with green and red lines, respectively

**Fig. 7:**
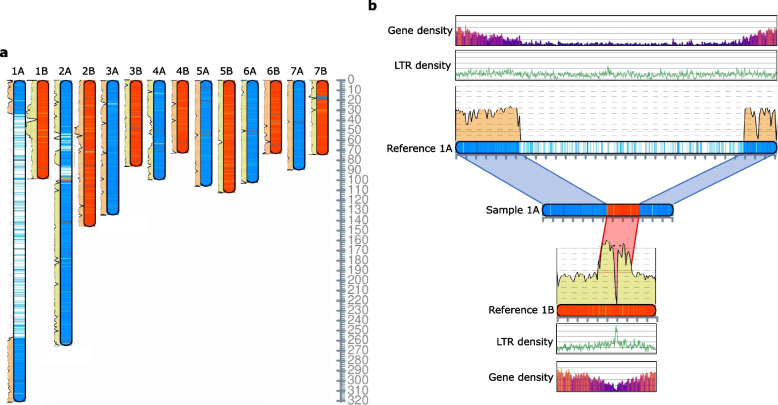
Sample ‘Arizona’ mapped to the *Poa annua* reference genome illustrates intraspecific variation in chromosome structure. **a** Depth of coverage is plotted in pastel alongside a cartoon representation of the *P. annua* reference genome. Sample ‘Arizona’ reads that preferentially map to the A and B subgenome parents are blue and orange, respectively. Orange segments within blue chromosomes and blue segments within orange chromosomes are putative HEs. Regions of the reference that have no reads mapped are white and indicate deletions. **b** The chromosome structures of the *P. annua* reference genome and sample ‘Arizona’ at chromosomes 1A and 1B. The 2 × increase in coverage at chromosome 1B signifies the presence of a large insertion, while the reduction in coverage at 1A signifies a deletion. Split reads at the deletion and duplication breakpoints show that the 1B duplication resides within the deleted region of the 1A chromosome. Gene and LTR density across the chromosomes indicate that the structural modification likely spans centromeric and pericentromeric sequences. The scale bar below chromosomes show hashes that are 10 Mb in length

Reads mapped to the *P. annua* reference genome (and diploid progenitor genomes) provide a view of structural modifications at the whole-chromosome level. Using this approach, we identified remarkable variation in chromosome structure post-polyploidization. The largest is a 224 Mb deletion in the centromeric and pericentromeric region of chromosome 1A in some samples that amounts to 70% of the length of the reference chromosome (Fig. 6; Fig. 7; Supplementary Fig. 14). Coinciding with the deletion at 1A is a 32 Mb duplication at chromosome 1B. Split reads and improperly paired reads at the deletion and duplication breakpoints suggest that the duplicated region at 1B resides within the deleted region of 1A, and indeed, capillary electrophoresis using homoeolog-specific markers across the chromosomal breakpoint confirms this to be the case (Supplementary Fig. 15). The 1B duplication contains the highest density of LTRs across the chromosome, suggesting that the rearrangement most likely spans the centromere (Fig. 7b). There are 1,996 annotated genes and 133 functional enrichments (mostly transposon-associated categories) in the 224 Mb centromeric deletion. The 32 Mb homoeologous centromere brings back 1,321 of the 1,996 deleted genes and all but four of the functionally enriched categories.

Perhaps the most parsimonious path to this karyotype involves meiotic error, where 1A and 1B form a quadrivalent and adjacent disjunction leads to two 1A’s going to one pole and two 1B’s going to the other. When fertilized by a normal nucleus, the resulting offspring would be 1A1B1B1B (or 1A1A1A1B). Subsequent generations would lead to introgression of 1A at recombination sites, which would cause most of the genic regions of the displacing 1B chromosome to return to a 1A-like state. Alternatively, it is possible that dysploidy and Robertsonian rearrangements (fusion-fission) played an intermediate role, where again, introgression back to the population resulted in the observed karyotype [56]. To our knowledge, cytological studies have not recorded any evidence of dysploidy in *P. annua*.

**Fig. 8:**
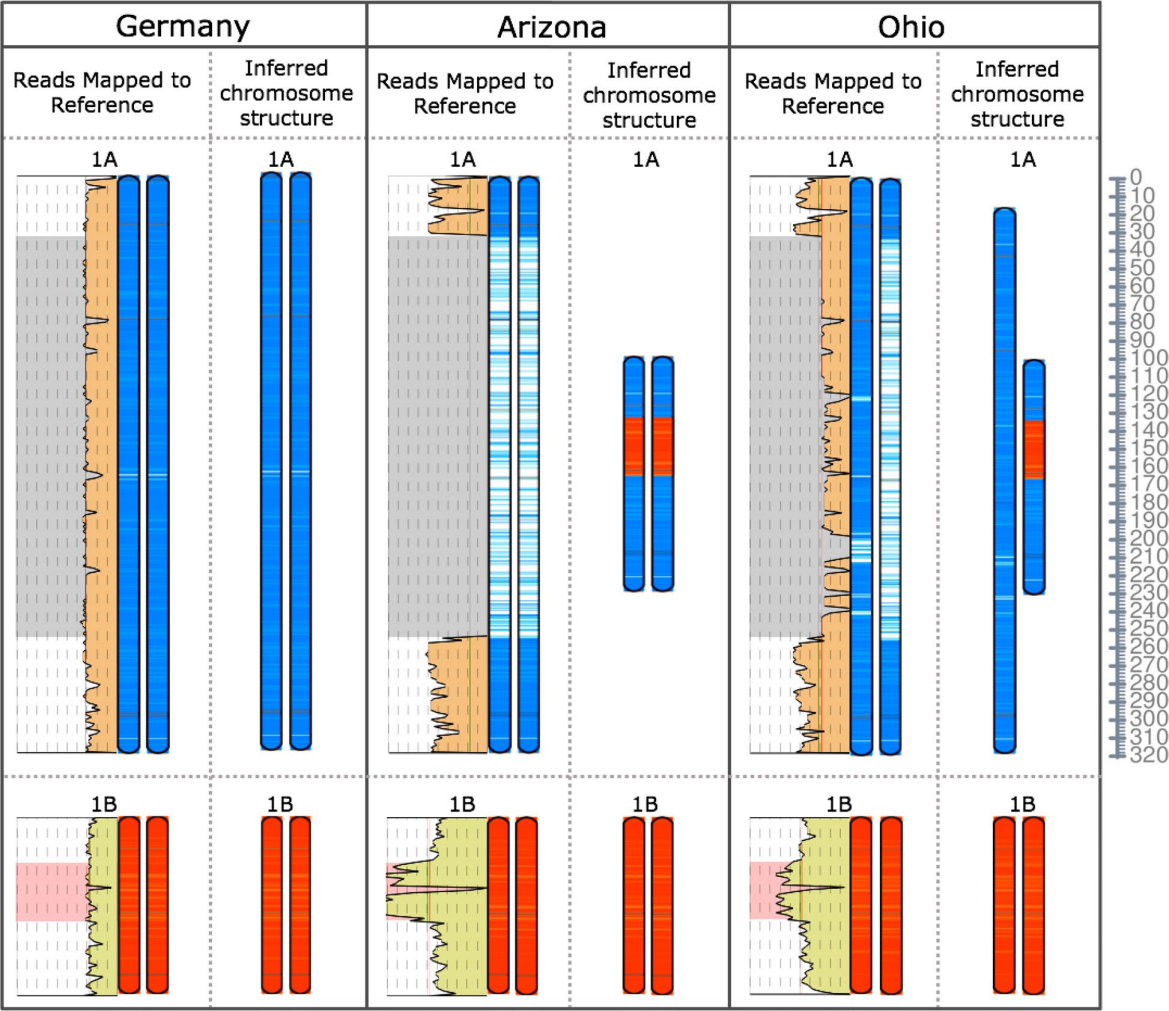
Sample reads mapped to the *Poa annua* reference genome depict intraspecific variation in chromosome structure. Depth of coverage is plotted along the chromosome. Regions where duplications and deletions occur are highlighted in pink and grey, respectively, and evident by depth of coverage that deviates from the mean. The pairs of cartoon chromosomes depict the two parental haplotypes of 1A (blue) and 1B (orange). The inferred chromosome structure of each sample is based on map coverage and coordinates of split-reads at breakpoint junctions. Sample ‘Ohio’ shows a haplotype-specific modification (heterozygote), likely originating from a cross between a Germany-like sample and an Arizona-like sample. The scale bar is in Mb

Chromosome 1A has more repetitive DNA (90%) than any other chromosome in the *P. annua* reference genome, which likely plays a role in the observed restructuring in some genotypes [57]. Most (99%) of the 224 Mb deleted region is low-complexity repetitive sequence, indicating that it would likely be wound into pericentromeric heterochromatin and suppressed from meiotic recombination [58–60]. Fittingly, rearrangements at 1A appear to reside at the periphery of heterochromatic sequences (Fig. 7b). Large-scale chromosomal rearrangements in *P. annua* occur in intragenic recombination ‘hotspots’ (resulting in a gene fusion between homoeologs), where individual genotypes display some variability in the exact nucleotide coordinates of the breakpoint but are often within several kilobases of each other (Supplementary Fig. 16; Supplementary Fig. 17). Some individuals appear to contain large-scale variability between their parental haplotypes (heterozygotes). For example, sample ‘Ohio’ has a copy of 1A that resembles the *P. annua* reference genome, while the other haplotype contains the 224/32 Mb centromeric displacement discussed above (Fig. 8; Supplementary Fig. 15). Such variability between haplotypes is surprising given that homologous chromosomes recognize each other by sequence similarity and incorrect pairing could lead to multivalents, which are associated with improper segregation and reduced fertility.

### Variation of EPSPS

*P. annua* is most commonly known as a noxious weed. It can be managed with both pre- and post-emergent herbicides, but repeated application has resulted in the evolution of multiple herbicide resistance pathways [61, 62]. Glyphosate resistance has been particularly problematic for managers of *P. annua.* Glyphosate works by inhibiting the enzyme 5-enolpyruvylshikimate-3-phosphate synthase (EPSPS) in the shikimate pathway. Resistant *P. annua’s* have been reported to have increased EPSPS copy number variation and missense mutations [63]. The A subgenome EPSPS gene is 3,891 bp in length, while the B subgenome copy is 9,092 bp. We identify large genotypic variation in the structure of EPSPS homoeologs, particularly in the B subgenome, where nearly 6 kb is deleted from the two largest introns in some genotypes (Supplementary Fig. 18). We do not see evidence of copy number variation in our resequencing data, but that is likely because none of our accessions originated from a population with known herbicide resistance.

## Discussion

### The plant cell’s response to WGD

Comparative genomics between the *P. annua* reference genome and the genomes of its diploid parents suggests that *some* tetraploid genotypes are remarkably unchanged since polyploidy. In contrast to paleo-allopolyploids where biased fractionation is a hallmark of diploidization, it appears that neo-allotetraploid *P. annua* is more accurately characterized by biased gene reshuffling, where the B (supina) subgenome has preferentially acquired genes from the A (infirma) subgenome in the absence of measurable loss. It is possible that reshuffling precedes fractionation, and if homoeolog expression bias and TE content are accurate early predictors of subgenome dominance, as is the case in other allopolyploids [52, 64, 65], the bias accumulator of genes (i.e., the B subgenome) will be preferentially retained (dominant).

### Retrotransposon response to WGD

The lifecycle of LTRs begins in the nucleus, gets transferred to the cytoplasm as mRNA, and re-enters the nucleus where it integrates into the genome (for review see Sabot and Schulman [66]). The location of LTR integration is at least partially stochastic and dictated by spatial nearness of susceptible host DNA upon re-entry into the nucleus. Our data suggests that the subgenomes of *P. annua* have varying ability to inhibit LTRs, both within and between subgenomes. Because inter-subgenome defense likely involves silencing LTRs after they re-enter the nucleus, we hypothesize that the observed bias in transposon movement from the A (infirma) subgenome to the B (supina) subgenome is partially driven by differences in the subgenome’s ability to repress retrotransposons post-transcriptionally. It is also likely that intrinsic properties of the A and B subgenomes, such as chromatin type (heterochromatin or euchromatin) and DNA methylation, play a role in susceptibility. We expect that newly formed allopolyploids with broadly divergent TE immunities will approach retrotransposon-equilibrium as the subgenome with fewer TE inhibitory mechanisms will be preferentially bloated by TEs.

### The plant cell’s response to retrotransposons in light of WGD

Although the *P. annua* reference genome closely resembles the parental genomes, this is not the case for all *P. annua* individuals, with some genotypes appearing heavily restructured relative to the genomes of the diploid progenitors. It is likely not a coincidence that the observed chromosomal rearrangements result in the substitution of a heavily TE-parasitized region with a less parasitized homoeologous segment. It appears that WGD has provided *P. annua* with the homoeologous ‘spare parts’ to purge highly parasitized sequences. This result supports the Genome Balance Hypothesis, which predicts that differences in the amount of pericentric heterochromatin between subgenomes (as observed between 1A and 1B) will cause chromosomes to move to the poles at uncoordinated times, and that the centromere of one of the parents will be retained to overcome those segregation issues [67]. We expect that our genome resequencing provides a snapshot of an organism caught in that act of positive selection for a balanced genome.

## Conclusions

*Poa annua* is a globally distributed neo-allotetraploid grass that benefits from the heterotic effects of having two distinct subgenomes, but our work shows that the homoeologs of *P. annua* have already begun to intermingle in meaningful ways. Purifying selection might slow the diploidization process but ultimately, we expect that *P. annua* will be exposed to the ecological consequences of subgenome homogenization and the inevitable functional divergence (neofunctionalization and subfunctionalization) and pseudogenization of duplicated alleles. It will be interesting to continue to unravel the aspects of the parental genomes that seem to have helped equip *P. annua* for success as an allopolyploid and to see if it can maintain its environmental foothold as one of the most prolific plants on earth over the next tens and hundreds of millennia as its genome continues to be modified.

Here, we present the chromosome-scale genome sequences of *P. infirma* and *P. supina,* the diploid progenitor species of allotetraploid *P. annua.* The genomic resources generated here, and in Robbins et al*.* [30], comprise one of the first reports detailing an allopolyploid and its progenitors sequenced to chromosome level. The insights into polyploid evolution that were generated as a result of this work have expanded our understanding of the relationship between plant homoeologs and TEs. Few species have the environmental versatility that *P. annua* has, and as such, the species serves as an appropriate model for studying biotic and abiotic stress tolerance in cereal crops and other agronomically significant polyploids. The genomic resources detailed in this work should serve as a powerful tool for turfgrass breeders and herbicide biologists to solve emerging agricultural challenges by facilitating better targeting of *P. annua’s* homoeologs and enabling the development of genetic markers that span chromosomal breakpoints for cost-effective surveys of chromosome architecture.

## Materials and methods

### Collecting genomic and transcriptomic resources

Seeds of *P. infirma* were obtained from the turfgrass breeding collection at the Pennsylvania State University and represent the only publicly available source of this species. The *P. infirma* accession used here was originally collected in Spain and acquired by Dr. Shui-zhang Fei at Iowa State University. The ‘Supranova’ cultivar was selected to represent *P. supina* as it is the most widely used cultivar on the market with agronomic application as a turfgrass, primarily known for its shade tolerance. Seeds were germinated on moist filter paper in petri dishes before being transferred to potting soil in a growth chamber at 20 °C and 8-h day lengths. A single genotype was selected for each species and clonally propagated by manually splitting plants at the basal meristem.

Genomic DNA was extracted from fresh leaf tissue using the cetyltrimethylammonium bromide (CTAB) method as outlined by OPS Diagnostics protocols with minimal vortexing and cut pipet tips to promote high molecular weight DNA extractions. Sample integrity was verified using pulsed-field electrophoresis and indicated an average size range between 50–70 kb. DNA was sheared to 20 kb length using a Megaruptor (PacBio). HiFi libraries were constructed using the PacBio Express kit, v2.0, and size selection was performed on a SageELF (Sage Science) to obtain narrow 15–20 kb libraries for sequencing using a PacBio Sequel II (Brigham Young University, DNA Sequencing Center). Three 8 M SMRT cells with 30-h movies were used for each diploid. PacBio sequencing yielded 72 Gb of Q20 reads for *P. infirma* (29 × fold coverage) and 45 Gb of Q20 reads for *P. supina* (30 × fold coverage)*.* For Omni-C proximity ligation (Dovetail Genomics), genomic DNA was re-extracted from the same genotypes after 72-h of dark treatment. One proximity ligation library was prepared for each species and sequenced using the Illumina HiSeq platform to obtain 464 million reads (28 × coverage) for *P. infirma* and 466 million reads (49 × coverage) for *P. supina* using 75 × 75 bp paired-end sequencing. We pooled a variety of tissues and treatment types for full-length RNA-sequencing with Iso-Seq (PacBio) to help facilitate high quality gene annotations. Tissue types included germinating seedlings, fresh leaves and root, and juvenile and mature inflorescences. Treatments included clonally propagated individuals that were exposed to 8-h light, 16-h light, cold (4 °C) treatment for two weeks, treated to 1″ simulated mowing stress for one week (five total cuts), and exposure to 100 mM NaCl for two weeks. Meristematic crown tissue was collected for each of the described treatments. All RNA samples were extracted using the Qiagen RNeasy Plant Mini Kit. RNAs for Iso-Seq were pooled and libraries were constructed using the PacBio express kit (v2.0). Each of the two Iso-Seq libraries per species ran for 24 h on an 8 M SMRT cells with a Sequel II instrument and yielded 4,026,288 million *P. supina* transcripts and 3,689,421 *P. infirma* full-length Iso-Seq transcripts.

### Nuclear genome assembly

K-mers were extracted from long-read (HiFi) sequencing data using Jellyfish (v2.2.10) [68] with 21-mers and a hash with 100 M elements (parameters ‘-m 21 -s 100 M’). GenomeScope (v.1) [69] was used to plot k-mers and estimate genome size, level of heterozygosity, and amount of repetitive sequence using 15,000 bp read lengths (Supplementary Fig. 19). K-mer analysis confirmed that *P. supina* was highly heterozygous and *P. infirma* was highly homozygous [47]. As a result, we selected different assembly pipelines for each species that best accommodated its unique biology. The genome of the highly heterozygous and obligate outcrosser, *P. supina,* was assembled with HiCanu (v2.1) [70] and purged to haplotig level using the Purge_Dups (v1.0.1) pipeline [71] with manual cutoffs adjusted according to its heterozygosity (calcuts parameters ‘-l 7 -m 40 -u 160’; minimum alignment score (-a) to 80). *Poa infirma* is self-pollinated and highly homozygous. As a result, we assembled the *P. infirma* genome using HiFiasm (v0.3) [72] with its built-in haplotype purging algorithm that is better suited for homozygous genome assemblies’. The Benchmarking Universal Single-Copy Orthologs (BUSCO; v3.0.2) software was used to estimate assembly completeness and their quality [73]. We also scanned for incorrectly placed centromeric and telomeric repeats using ‘bedtools nuc’ and a 1 Mb sliding window to count the occurrences of common repetitive sequences found in the centromeric and telomeric sequences of Poaceae. The purged haplotype assemblies and raw Omni-C reads were input into the HiRise pipeline (Dovetail Genomics) to scaffold contigs, identify chimeric scaffolds, and build a final genome assembly based on proximity ligation (Supplementary Fig. 1). Taxonomic classification with Kraken2 (v2.1.1) [74] was used to filter out potential contaminants from the final assemblies and verify that the chromosomes did not contain non-plant DNA, which may indicate a chimeric assembly. The *P. infirma* genome assembled into seven pseudomolecules and 873 supplementary scaffolds. The *P. supina* nuclear genome contained seven pseudomolecules and 357 supplementary scaffolds. *Poa supina* pseudomolecules ranged between 73 and 115 Mb, while *P. infirma* ranged between 90 and 331 Mb in length. The seven chromosomes of each species were re-oriented, if necessary, to reflect identical strand orientation across all pairs of orthologous chromosomes. Chromosomes were renamed according to pre-established chromosomal nomenclature and large structural modifications between each diploid and the allotetraploid *P. annua* were verified by sequence alignment using minimap2 with parameters ‘–secondary = no -cx asm10’ (v.2.24) [75].

### Chloroplast genome assembly

Raw whole-genome sequenced HiFi reads were mapped to the *P. annua* chloroplast reference genome (GenBank acc: NC_036973.1) using minimap2 (v2.24) using ‘map-hifi’. Samtools (v1.9) [76] was used to identify mapped reads with a minimum query length (mlen) > 8000, query value (qval) > 60 and GC content between 32 – 52%. Reads with a length > 20,000 bp were then included in a final de novo assembly of the chloroplast genome with HiCanu (v2.1) using default parameters. A circular genome was predicted by HiCanu, which was subsequently trimmed as projected by HiCanu at the same starting point as the reference chloroplast genome. Sequence alignment of the circular chloroplast genomes for each species verified that *P. infirma* is the maternal parent to *P. annua* (Supplementary Fig. 20)*.*

### Repeat masking and LTR insertion times

De novo repeat libraries were created for each diploid assembly using the Dfam (v3.1) [77] database to classify transposable DNA sequences. RepeatModeler (v2.0.3) [78] with the parameter ‘-LTRStruct’ was used to model TE family relationships and identify repetitive elements by employing programs RECON, RepeatScout, LTRHarvest [79] and LTR_retriever (v2.8.7) [80]. The resulting TE consensus classification libraries were used as input into RepeatMasker (v4.1.2) to softmask each of the genome assemblies using the wublast engine. LTR_FINDER_parallel (v1.1; with parameter ‘-harvest_out’) [81] and LTR_retriever were run separately on all three species to calculate the insertion times for intact LTR elements. A rice mutational rate of 1.3 × 10^–8^ substitutions per year was used to calculate insertion times using the formula T = K/2µ, where K is the divergence rate calculated based on LTR sequence identity and µ is the neutral mutational rate in mutations per bp per year [82].

### Genome annotation

RNA-sequencing runs SRR1634026 and SRR1634028 were downloaded from NCBI’s Sequence Read archive database representing *P. supina* and *P. infirma*, respectively. *Poa annua* sequences from experiments SRR1634028, SAMD00020897, and SAMD00020898 were also acquired. All NCBI sequencing experiments were then trimmed for adapter content and low quality using bbduk with ‘tbo tpe ktrim = r k = 23 mink = 11 hdist = 1’. Cleaned reads from NCBI could be larger than 20 gigabytes, so we randomly subset each experimental run into a single 400-megabyte file. Each fastq file was aligned to the respective genome using the splice-aware algorithm, HISAT2 (v2.2.1) [83]. Iso-Seq transcripts for each species were aligned using minimap2 with ‘-ax splice:hq -uf’. NCBI and Iso-Seq alignment files were sorted by name and converted to bam format. The OrthoDB plant protein database (v10) was downloaded and expanded to include amino acid sequence annotations from the Poales available through NCBI refseq and Uniprot TrEMBL. BRAKER2 (v2.1.5) was run in ETP mode to incorporate both the enhanced OrthoDB protein data and the RNA alignment data from NCBI and Iso-Seq to train GeneMark-ETP with proteins processed by ProtHint. Augustus was trained based on the GeneMark-ETP predictions and the resulting protein predictions were hints from both sources. BRAKER2 added 5’ and 3’ UTRs using ‘–addUTR = on’ to call GUSHR. Annotations were filtered using sequence similarity to orthologous groups and phylogenies in the eggNOG [84] database (v2.0.5) using diamond alignments to retain only those annotations with fine-grained orthologous relationships. BUSCO (v3.0.2) was used in transcriptome mode to identify the majority (96% and 91%) of the 1,614 conserved embryophyta_odb10 orthologs were present in our *P. infirma* and *P. supina* chromosome annotations*,* respectively, supporting high-quality genome annotations. Long noncoding RNAs were identified using RNAplonc (v1.1) [85] that uses a classifier approach developed specifically for plants. The chloroplast genome assemblies were functionally annotated using GeSeq [86].

### Cytology

C-banded chromosome preparations were made from root-tip meristematic cells according to the protocol described by Mitchell et al*.* [87] except that 0.02% colchicine, not trifluralin, was used to arrest microtubule formation for 2–4 h at room temperature.

### RNA-seq expression analysis and homoeolog expression bias

Plants were collected from an ongoing field trial from the turfgrass breeding program at the Pennsylvania State University. For each *P. annua* breeding line, at least one typical dwarf-type and one aberrant wild-type plant were collected from a genetically pure unmowed stand. Dwarf-types were defined as any genotype with diameter ≤ 1.5 cm, while aberrant wild-types had a diameter ≥ 6 cm. Plants were transplanted to a greenhouse (27 °C high and 17 °C low) and clonally propagated over two months. To simulate mowing treatment, one clone of each genotype was trimmed three times per week and maintained at 1.5 cm height and the other clone was left untrimmed. The experiment was conducted on 30 plants representing 15 unique genotypes (six dwarf-types and nine wild-types). Spacing on the bench was randomly assigned. Treatments were applied between May 10^th^ and August 16^th^, 2020. All plants were allowed to grow unmowed for an additional three weeks prior to tissue collection to reduce the influence of wounding stress in our data analysis. Tissue was collected from the grass’s basal meristem.

Unique libraries for each sample were created using the Lexogen SENSE mRNA-seq library kit with the goal of producing long insert sizes of ~ 485 bp for simplified and accurate inference of parental origin across homoeologous pairs [88]. A pilot study was conducted using a MiSeq with Nano kit reagents (v2) to obtain 500 Mb of 250 × 250 bp paired-end sequencing. The pilot analysis revealed that insert sizes were generally shorter than anticipated with 75% of inserts being ≤ 260 bp. Adjusting for shorter library lengths, we sequenced the RNAs using an S1 flow cell on an Illumina NovaSeq 6000 (Pennsylvania State University, Genomics Core Facilities) to obtain 150 × 150 bp paired-end reads with ~ 48 million reads/sample.

We used Eagle-RC (v1.1.2) [89] to classify RNA-seq reads to their appropriate subgenome using explicit genotypic differences between them to calculate the likelihood that an RNA read came from a particular subgenome. Briefly, variant candidates for statistical inference were generated using reciprocal LAST (v1387) [90] to identify homoeologous genes and an Eagle-RC python script (homeolog_genotypes.py) to create the variant file (VCF). Reads were mapped to the parental genomes separately using STAR (v2.7.8a) [91]. The EAGLE model subsequently evaluated the likelihood of each reads subgenome origin based on genotypic variants and assigns a likelihood score. Alignments with SNP evidence to support subgenome origin are dubbed homoeolog-specific and quantified with featureCounts (v2.0.2) [92]. The resulting counts matrix was filtered to retain only the genes that had at least one read per sample. The ‘run_DE_analysis.pl’ script from the trinityrnaseq toolkit (v2.13.0) [93] was used to run DESeq2 (v1.38) [94] on the counts matrix for subgenome-specific differential expression analysis. Because plant biotypes (dwarf or wild) were nested within treatments (mowed or unmowed), biotype as a variable was removed to prevent erroneous interpretation in our mowed vs unmowed and A vs B subgenome comparisons (Supplementary Fig. 9).

### Gene ontology enrichment analysis

Gene ontologies and functional enrichments of differentially expressed genes were classified using the Trinotate pipeline. Blastp and Blastx (v2.12) [95] were used to align *P. annua* amino acids and coding sequence files against the Uniprot Swissprot database with parameters ‘-max_target_seqs 1 -outfmt 6 -evalue 1e-3’. Hmmscan (v3.3.2) was used to incorporate protein domain identification based on query against the pfam database. An id2go formatted file was then generated using the blastx, blastp, and hmmscan results to incorporate the Swissprot and pfam alignments using go-basic and pfam2go annotations from geneontology.org. The id2go formatted file was incorporated into ‘analyze_diff_expr.pl’ (trinityrnaseq toolkit) with the ‘–examine_GO_enrichment’ flag to call the R package Goseq to scan for enriched gene ontologies in our subgenome-specific differential expression matrix. The id2go formatted file was also used as input into Goatools script ‘find_enrichments.py’ [96] to identify enriched ontologies in various subsets of genes of interest. Candidates from enriched subsets were further analyzed using EggNOG-mapper and BLAST for functional annotation at the single-gene level.

### Comparative genomics

*P. annua* (PaA & PaB) and a concatenated file containing the diploid parents (PiA & PsB) were uploaded into CoGe SynMap tool [97] with DAGChainer options ‘-D 20 -A 5’ and tandem duplication distance set to 10. Synonymous mutation (Ks) was calculated on the syntenic CDS pairs using CodeML of the PAML package. Ks values were plotted on a density plot to visualize Ks peaks associated with parental divergence and hybridization. For CoGe’s fractionation bias calculation, syntenic blocks were merged using the ‘Quota Align Merge’ algorithm with a maximum distance between two genes (-Dm) set to 40. Syntenic depth was calculated with the ‘Quota Align’ algorithm and ratio of coverage depth set to 1-to-2. The window size for fractionation bias was adjusted to 100 genes and set to only use syntenic genes in the target genome. MCScanX [98] was used to detect syntenic blocks of genes between *P. annua* and the diploid progenitors. The collinear file was input into SynVisio [99], an interactive multiscale synteny visualization tool to depict regions of shared homology. Syntenic pairs and macrosynteny in monocots was calculated using the MCscan (python version) with *Ananas comosus* and *Brachypodium distachyon* coding sequences and genomes downloaded from Phyotozome (v12) [100]. A C-score of 0.99 was used to select only 1:1 orthologous blocks and is stringent enough to filter out syntenic blocks that were not LAST reciprocal best hit. Translated transcriptomes of model grasses were acquired through Phytozome and were asigned into orthogroups using Orthofinder with the Diamond algorithm for similarity searches. Average nucleotide identity (ANI) was calculated using the gap-compressed per-base sequence divergence output (de tag) of a PAF formatted full genome assembly alignment using minimap2. DupGen_finder [42] was used to identify single-gene duplicate pairs and classify them as either WGD, tandem, proximal, transposed, or dispersed. A concatenated fasta file containing both parental diploids (PiA & PsB) was used as an outgroup so that the transposed classification included only those genes that were duplicated after the hybridization of *P. annua.*

### Identification of HEs

HEs regions were characterized using several different methods to assure accurate identification. First, CNVkit (v0.9) [101] was used to identify and visualize copy number variants by mapping HiFi (ccs) reads from *P. annua* onto the parental diploid genomes (PiA & PsB). Second, minimpa2 with parameter ‘-x map-hifi’ was used to map *P. annua* HiFi reads to the concatenated fasta containing the parental diploid genomes (PiA & PsB). The resulting bam file was input into SVIM (v1.0.2) [102] and used to detect structural variants from our long-read sequencing data and extract split-reads with translocation breakpoints, called BNDs by SVIM. Split-reads were extracted from the bam file and used to detect beginning and endpoint of a HE block. Thirdly, we used mmseqs [103] with parameters ‘easy-rbh -s 7.5’ to identify *P. annua* coding sequences with reciprocal best hits corresponding to the other subgenome (PaA genes with RBHs on PsB or PaB genes with RBHs on PiA). Finally, we used a primary mapping approach where *P. annua* HiFi reads are aligned to a fasta file containing both parental diploid genome (PiA & PsB). Reads with primary mapping flag were retained and sorted into two pools, reads that mapped to the PiA genome and reads that mapped to the PsB genome. Both pools of *P. annua* reads assigned a custom tag based on their parental mapping and were re-mapped to *P. annua*. If a *P. annua* read mapped best to the *P. infirma* (PiA) parent but subsequently mapped to *P. annua’s* B (supina) subgenome, it was a candidate for HE. All four HE methods were compared and it was determined that the primary mapping approach was superior as it was visually verifiable in the Integrative Genomics Viewer (IGV) [104] and produced HE statistics that were most intermediate to the other methods. The JCVI chromosomal painting tool (jvci.graphics.chromosome) was used to visualize *P. annua’s* HEs.

### Resequencing *P. annua*

15 geographically distinct *P. annua* accessions were sequenced to survey genotypic variation across the species. *Poa annua* is a heavily admixed species, and as such, it is difficult to know the degree to which accessions are genetically isolated from each other. Our goal was to choose 15 accessions across distinct locations to maximize our chances of capturing a wide range of genetic diversity. Samples ‘Germany’ (W6 28,152), ‘Nunavut’ (PI 236900), ‘India’ (PI 217625), and ‘Belgium’ (PI 442543) were acquired from the Germplasm Resources Information Network (GRIN) through the US Department of Agriculture. Samples ‘Washington’ (Tacoma), ‘Scotland’ (Galloway), ‘New Zealand’ (Manawata), ‘Arizona’, ‘Quebec’, ‘Wales’ (Aberystwyth), ‘Sweden’ (Särö), ‘New York’ (Pa-33) and ‘Ohio’ (Columbus) were acquired from a breeding collection maintained at the Pennsylvania State University. Seeds were germinated on moist filter paper. A single genotype of each of the thirteen samples was transferred to potting soil (Promix) and grown in greenhouse. In addition to the thirteen geographically distinct accessions, two breeding lines were included. ‘Pa-14 dwarf’ and ‘Pa-14 wild-type’ are derived from the same breeding pedigree of an unstable line (Pa-14), where ‘wild’ describes an aberrant wild-type plant and ‘dwarf’ describes an agronomically desirable dwarf individual. DNA was extracted from all 15 samples using fresh leaf tissue and the CTAB method as described above. Plants were genotyped to confirm that they were authentic *P. annua’s* using the Trx2 nuclear gene with PCR parameters described in Mao and Huff [18], and Patterson et al*.* [105]. Genomic DNA (300 ng) from each sample was input into the Illumina DNA PCR-Free Prep kit to create uniquely indexed libraries. The samples were pooled and an equimolar concentration was verified using a MiSeq Nano 150 × 150 bp. The pooled sample was sequenced on a NovaSeq S1 (Pennsylvania State University, Genomics Core Facilities) with 150 × 150 bp paired-end sequencing to generate a target of 1.3 – 1.6 billion pairs and 15–20 × coverage per genotype across the haploid (1.78 Gb in size [30]) genome.

Raw Illumina reads were trimmed for adapter sequences using bbduk as described above and aligned to the *P. annua* reference genome using bwa-mem2. Scaffolds corresponding to the chloroplast and mitochondrial genomes were included in the *P. annua* reference genome to prevent erroneous alignment of plastid sequences to the genome. Coverage across chromosomes and scaffolds were plotted using WGSCoveragePlotter.jar (jvarkit). Putative HEs were annotated similarly as described above. Briefly, raw reads were mapped to a file containing the parental *P. infirma* (A) and *P. supina* (B) genomes. Primary alignments were tagged according to their parental origin and re-mapped to the *P. annua* reference genome. Reads that mapped to a different parental genome than *P. annua* subgenome were potentially a HE. We then classified each coordinate in the *P. annua* reference as either derived from *P. supina*, derived from *P. infirma*, or novel (not derived from either parent). In contrast to the HE pipeline used above that used HiFi (ccs) data, novel regions were annotated independently as opposed to being unincluded in the bed file. This adjustment allowed more accurate visualization of short-reads with jcvi.graphics.chromosome.

Presence-absence variants were analyzed using an SGSGeneloss-based protocol, described in Fernandez et al*.* [106]. Illumina reads were mapped to the parental genomes and subsequently the *P. annua* genome to identify HE regions as described above. The alignment file for each sample was converted to bed format using bamToBed from bedtools (v2) [107]. The alignment bed was merged with the gene annotation file using bedtools intersect to identify regions of overlap. If a gene’s coordinates contained < 20% coverage in the sample, it was deemed lost (dispensible) in that sample. If it had > 90% coverage in the opposite parent, it was deemed a gene within an HE.

Large-scale structural variants were annotated manually by analyzing the depth of coverage of each sample alignment to both progenitor and allotetraploid genomes. Duplicated and deleted sequences will cause deviation from the mean and median coverage. Duplicated sequences align to the next most homologous coordinates in the reference genome and are visible by elevated coverage at that site. Deleted sequences are detectible by reduced coverage at the missing region. Transposed sequences are represented in equal proportion in the reference and in the sample, therefore they do not cause deviations from the mean coverage. Split reads and improperly paired reads at the junctions of duplicated and deletion breakpoints can be used to further specify the exact coordinated of the exchanges. Commonly used tools that identify structural variants such as Delly2, Manta, and Lumpy are not equipped to identify insertions larger than several kilobases in length. Large-scale structural rearrangements were verified using homoeolog-specific primers and Sanger sequencing. Primer pair 1AF (5′- GGCGGACACCTTTGACACC) and 1AR (5′- GGATACTCAGACAATGATAG) amplify using standard PCR settings with a 53 °C annealing temperature and 1:00 extension time. Primer pair 1AF (5′- GGCGGACACCTTTGACACC) and 1BR (5′- GGGTGACAGAGTTCCCAGTG) amplify using standard PCR settings with a 65 °C annealing temperature and 1:20 extension time. 1AF to 1AR spans a chromosomal breakpoint and only amplifies in the absence of the 32/224 Mb structural modification. 1AF to 1BR spans the same chromosomal breakpoint but only amplifies in the presence of a rearrangement (Supplementary Fig. 15).

SNPs were identified from each of the 15 samples using their corresponding *P. annua* alignment file. Picard MarkDuplicates was used to tag duplicated reads and reduce the frequency of incorrect SNP calls. The duplicate-marked bam files were used to generate genotype likelihood calls across all samples and chromosomes using parameters ‘-q 40 –ff UNMAP,SECONDARY,QCFAIL,DUP’ with bcftools mpileup and subsequently input into bcftools call with default parameters. Variants were further filtered with vcftools using parameters ‘–remove-indels –maf 0.1 –max-missing 0.9 –minQ 30 –min-meanDP 10 –max-meanDP 80 –minDP 10 –maxDP 80’.

## Supplementary Information

**Additional file 1:** **Supplementary Table 1.** Features of the genome assemblies and annotations. Parental species, *Poa infirma *(PiA) and *P. supina *(PsB), relative to the subgenomes of *P. annua *(PaA and PaB). Values correspond to the seven pseudomolecules. BUSCOs are *n*=1,614. **Supplementary Fig. 1.** Linked density histograms of the *Poa infirma* and *P. supina* genomes. The Omni-C plot represents long-range *cis *information using proximity ligation and mapping position of paired-end data. Red indicates the number of read pair interactions within each bin. **Supplementary Fig. 2.** The homoeologous and orthologous sequences of *Poa annua *and its diploid parents, *P. infirma *and *P. supina. *(a) The evolutionary pathway and chromosomal relationships within and between the homologous sequences of *P. annua *and its diploid progenitors. (b) The distribution of sequence identity measured by the gap-compressed sequence identity of full-genome alignments. Percentages above violin plots indicate the median. (c) Chromosome lengths of the genome assemblies of all three species. **Supplementary Fig. 3.** Whole-genome sequence alignment depicts the primary mapping of parental chromosomes (PiA & PsB) to allotetraploid, *Poa annua *(PaA & PaB). The black arrow over Pa2B highlights 30 Mb of novel sequence in the tetraploid genome assembly, mostly composed of repetitive DNA (22.7Mb). **Supplementary Fig. 4. **The distribution of shared orthologous clusters (gene families) between the A and B genomes of *Poa infirma *(PiA)*, P. supina *(PsB)*, *and *P. annua *(PaA & PaB)*. *Single-copy gene clusters are not depicted. **Supplementary Fig. 5.** Estimated molecular divergence of the PaA and PaB subgenomes of *P. annua *and the genomes of the diploid parents (PiA and PsB). Arrows and corresponding values highlight the peak density of synonymous substitutions. **Supplementary Fig. 6.** Sequence alignment of five model monocots spanning two whole-genome duplications. (a) A phylogenetic tree shows the species relationships. Pineapple (*Ananas comosus*) serves as outgroup because its speciation from the BOP and PACMAD clades of grasses predates the most recent of the ancestral Poaceae WGD events, rho (r). (b) Genomic alignment between monocots *Ananas comosus, Brachypodium distachyon, Poa infirma, P. supina, *and *P. annua*. Percentages in red show the ratio of 1:1 orthogroups relative to *A. comosus*. The syntenic block highlighted in green shows the colinear evolution of a cluster of genes. **Supplementary Fig. 7.** Retention of *Poa annua *genes across the parental chromosomes of *P. infirma *and *P. supina. *PiA to PaA and PsB to PaB have elevated gene retention (~97%) across chromosomes and reflects minimal gene loss (fractionation) since polyploidization. PiA to PaB and PsB to PaA have lower gene retention (~61%) and reflects the quantity of genes retained since the two parental lineages diverged from their common ancestor. The red arrow highlights the largest homoeologous exchange in the *P. annua *genome. **Supplementary Fig. 8.** Principal component analysis compares the gene expression profiles of 30 *Poa annua *samples. On the right, a PCA including all samples, with variables being subgenome (A or B), treatment (mowed or unmowed), and biotype (dwarf-type or wild-type). On the left, a PCA of the A subgenome illustrates that samples cluster by mowing treatment but biotypes are nested. **Supplementary Fig. 9.** Differential gene expression analysis across the A and B subgenomes of *Poa annua. *(a) Principal component analysis of the differential gene expression profiles of *P. annua *samples with biotypes (dwarf-type or wild-type) removed from the analysis. (b) A heatmap of the DEGs across subgenome and treatment. Blue genes are upregulated and orange are downregulated. (c) Clusters of genes with similar expression profiles. On the left, a cluster of genes upregulated in the B subgenome. On the right, a cluster upregulated in unmowed plants. In the DEG hierarchal cluster and dendrogram, red=B_mowed, green=B_unmowed, purple=A_unmowed, blue=A_mowed. **Supplementary Fig. 10.** Treemaps cluster enriched gene ontologies in the subgenome (A vs B) and treatment (mowed vs unmowed) comparisons based on semantic similarity of enriched terminologies. **Supplementary Fig. 11.** Geographic distribution of 13 re-sequenced *Poa annua *accessions. Two additional plants (PA-14 dwarf and Pa-14 WT) were also sequenced but not depicted on the map. **Supplementary Fig. 12.** SNP density across a 1 Mb sliding window demonstrates variability in sequence identity between *Poa annua *accessions and across chromosomes*. *Pink is sample ‘Arizona’ and blue is sample ‘Wales’. Coverage plots of both samples are included for reference. The scale bar is 320 Mb in length with each hash representing 10 Mb. **Supplementary Fig. 13.** The core *Poa annua *genome. Of the 76,541 gene annotationsin the *P. annua *reference genome, 68,733 are present in all 15 re-sequenced genotypes. 7,808 genes are dispensable and absent in at least one genotype. **Supplementary Fig. 14. **Depth of coverage plotted along the *Poa annua *reference genome suggests large-scale structural modification in *P. annua *chromosomes. **Supplementary Fig. 15.** Homoeolog-specific markers and Sanger sequencing verifies the composition of a large-scale chromosomal rearrangement in *Poa annua*. Alignment of the homoeologous sequences of Pa1A and Pa1B span the breakpoint of a large-scale chromosomal rearrangement in re-sequenced genotype ‘Arizona’ but not ‘Germany’. Sample ‘Ohio’ has both a rearranged and non-rearranged chromosome, verifying the haplotype specificity (heterozygotes) of chromosome rearrangements in some individuals. Black arrows highlight homoeolog distinguishing SNPs on either side of the breakpoint. **Supplementary Fig. 16.** Sequence alignment of three samples to the *Poa annua *reference genome illustrates recombination hotspots. Arrows point to alignment breakpoints at the 224 Mb deletion. Black arrows point to breakpoints that occur on the same sequence coordinates for both parental haplotypes. Red arrows point to haplotype-variable breakpoints. Blue boxes at the top of the alignment window show genes with exons (boxes) and introns (lines connecting boxes). Arrows that are perpendicular to genes are gene fusion events. **Supplementary Fig. 17.** Sequence alignments at chromosome 1A illustrates local variability at crossover ‘hotspots’. Black arrows indicate positions where both pairs of homologous chromosomes break at the same location and red arrows point to haplotype-variable breakpoints. Blue boxes at the top of the alignment window show genes with exons (boxes) and introns (lines connecting boxes). Arrows that are perpendicular to genes are gene fusion events. **Supplementary Fig. 18.** Sequence alignment of four *Poa annua *accessions shows structural variation at its two EPSPS homoeologs. ajg15317 and ajg73723 are EPSP synthases on chromosomes Pa5A and Pa5B, respectively. Blue boxes at the top depicts the genes exons (boxes) and introns (lines connecting boxes). The ajg15317 transcript is 3,891 bp in length, while ajg73723 is 9,092 bp. Grey boxes are reads that aligned to the reference genome as proper pairs. Open boxes are reads that mapped equally well to five or more locations in the genome. Red pairs have longer than anticipated insert lengths and depict putative indels at ajg73723’s longest introns. Sample ‘Sweden' is heterozygous for a 2,738 deletion at the second intron, while ‘Wales’ and breeding line ‘Pa-14 dwarf’ are homozygous for the deletion. Only the breeding line sample, ‘Pa-14 dwarf’ contained a 2,954 deletion at the 7^th^ intron. Purple alignments in ajg15317 show reads with mates that map to the other subgenome homoeolog (ajg73723), within the indel at the second intron.** Supplementary Fig. 19.** Linear K-mer profiles and fitted models of the *Poa infirma *and *P. supina *genomes*. *Black lines show the fit of the model to the distribution of K-mer frequencies (blue). Sequencing errors are identified by low coverage k-mers shown in orange. The *P. infirma *and *P. supina *models follow a diploid distribution with low and high heterozygosity, respectively. **Supplementary Fig. 20.** The chloroplast sequences of *Poa infirma *and *P. supina. *(a) Chloroplast maps for *P. supina *and *P. infirma*. (b) Sequence alignment of chloroplasts show that *P. annua’s *maternal parent is *P. infirma.*(PPTX 45713 KB)

## Data Availability

Genome assembly and gene annotation files are available through the CyVerse CoGe platform and the International Weed Genomics Consortium (IWGC) WeedPedia platform, hosted by KeyGene. Raw sequence data are available in the Sequence Read Archive under NCBI BioProject PRJNA938153. Relevant plant resources are accessible through the Germplasm Resources Information Network and through the Pennsylvania State University Turfgrass Breeding Program Repository. This research complies with relevant institutional, national, international, and legislative guidelines relating to the handling of wild and cultivated plant materials.
