# Correction: Homoeologous evolution of the allotetraploid genome of *Poa annua* L

**DOI:** 10.1186/s12864-023-09510-2

**Published:** 2023-07-18

**Authors:** Christopher W. Benson, Matthew R. Sheltra, Peter J. Maughan, Eric N. Jellen, Matthew D. Robbins, B. Shaun Bushman, Eric L. Patterson, Nathan D. Hall, David R. Huf

**Affiliations:** 1 Department of Plant Science, Pennsylvania State University, University Park, PA USA; 2 Intercollegiate Graduate Degree Program in Plant Biology, Pennsylvania State University, University Park, PA USA; 3 Department of Plant and Wildlife Sciences, Brigham Young University, Logan, UT USA; 4 USDA ARS, Forage and Range Research, Logan, UT USA; 5 Department of Plant, Soil, and Microbial Sciences, Michigan State University, East Lansing, MI USA

**Correction: BMC Genomics 24, 350 (2023)**

**https://doi.org/10.1186/s12864-023-09456-5**

Following publication of the original article [1], it was reported that there was an error in Fig. 2, namely that the colour was missing from the colour scale. The correct Fig. 2 is given in this Correction and the original article has been updated.

**Fig. 2:**
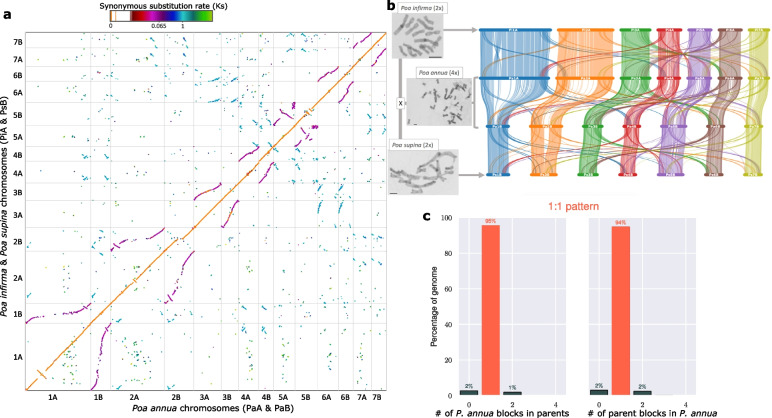
The comparative colinear relationship of three *Poa* genomes*.* **a** Macrosyntenic comparison of the *P. annua* genome (PaA & PaB; x-axis) to the combined *P. infirma* (PiA) and *P. supina* (PsB) genomes (y-axis). PiA to PaA and PsB to PaB comparisons are orange. Breaks in the contiguity of the orange line illustrate recent structural modifications occurring after the hybridization of the tetraploid (post-polyploidy). PiA to PaB and PsB to PaA comparisons are purple and illustrate structural modifications that occurred after the parental diploid species diverged from their common ancestor. The ‘S’ curve in some syntenic comparisons illustrates differences in chromosome size. Colors denote synonymous substitution rate (Ks). The Ks values in the scale bar indicates three important events: polyploid hybridization (Ks = 0), speciation of the parents (Ks = 0.065), and the rho (ρ) WGD event (Ks = 1). **b** The photomicrograph and syntenic ribbon plot depict the relative chromosome size, structure, and collinear relationship between the genomes of the *P. infirma, P. supina,* and allotetraploid *P. annua.* Scale bar in the bottom corner of the photomicrograph indicates the relative chromosome sizes. **c** The ratio of syntenic depth between genes of the diploid parents and genes of *P. annua* indicate a 1:1 relationship

## References

[1] Benson CW, Sheltra MR, Maughan PJ (2023). Homoeologous evolution of the allotetraploid genome of *Poa annua* L. BMC Genomics.

