# Supplementary material for: Homoeologous evolution of the allotetraploid genome of Poa annua L
Source: BMC Genomics. 2023 Jun 26;24:350. doi: 10.1186/s12864-023-09456-5 (PMC10291818; doi:10.1186/s12864-023-09456-5)
Supplement: Supplementary file 1 — Additional file 1: Supplementary Table 1. Features of the genome assemblies and annotations. Parental species, Poa infirma (PiA) and P. supina (PsB), relative to the subgenomes of P. annua (PaA and PaB). Values correspond to the seven pseudomolecules. BUSCOs are n=1,614. Supplementary Fig. 1. Linked density histograms of the Poa infirma and P. supina genomes. The Omni-C plot represents long-range cis information using proximity ligation and mapping position of paired-end data. Red indicates the number of read pair interactions within each bin. Supplementary Fig. 2. The homoeologous and orthologous sequences of Poa annua and its diploid parents, P. infirma and P. supina. (a) The evolutionary pathway and chromosomal relationships within and between the homologous sequences of P. annua and its diploid progenitors. (b) The distribution of sequence identity measured by the gap-compressed sequence identity of full-genome alignments. Percentages above violin plots indicate the median. (c) Chromosome lengths of the genome assemblies of all three species. Supplementary Fig. 3. Whole-genome sequence alignment depicts the primary mapping of parental chromosomes (PiA & PsB) to allotetraploid, Poa annua (PaA & PaB). The black arrow over Pa2B highlights 30 Mb of novel sequence in the tetraploid genome assembly, mostly composed of repetitive DNA (22.7Mb). Supplementary Fig. 4. The distribution of shared orthologous clusters (gene families) between the A and B genomes of Poa infirma (PiA), P. supina (PsB), and P. annua (PaA & PaB). Single-copy gene clusters are not depicted. Supplementary Fig. 5. Estimated molecular divergence of the PaA and PaB subgenomes of P. annua and the genomes of the diploid parents (PiA and PsB). Arrows and corresponding values highlight the peak density of synonymous substitutions. Supplementary Fig. 6. Sequence alignment of five model monocots spanning two whole-genome duplications. (a) A phylogenetic tree shows the species relationships. Pineapple (Anana [file 12864_2023_9456_MOESM1_ESM.pptx]

## Slide 1
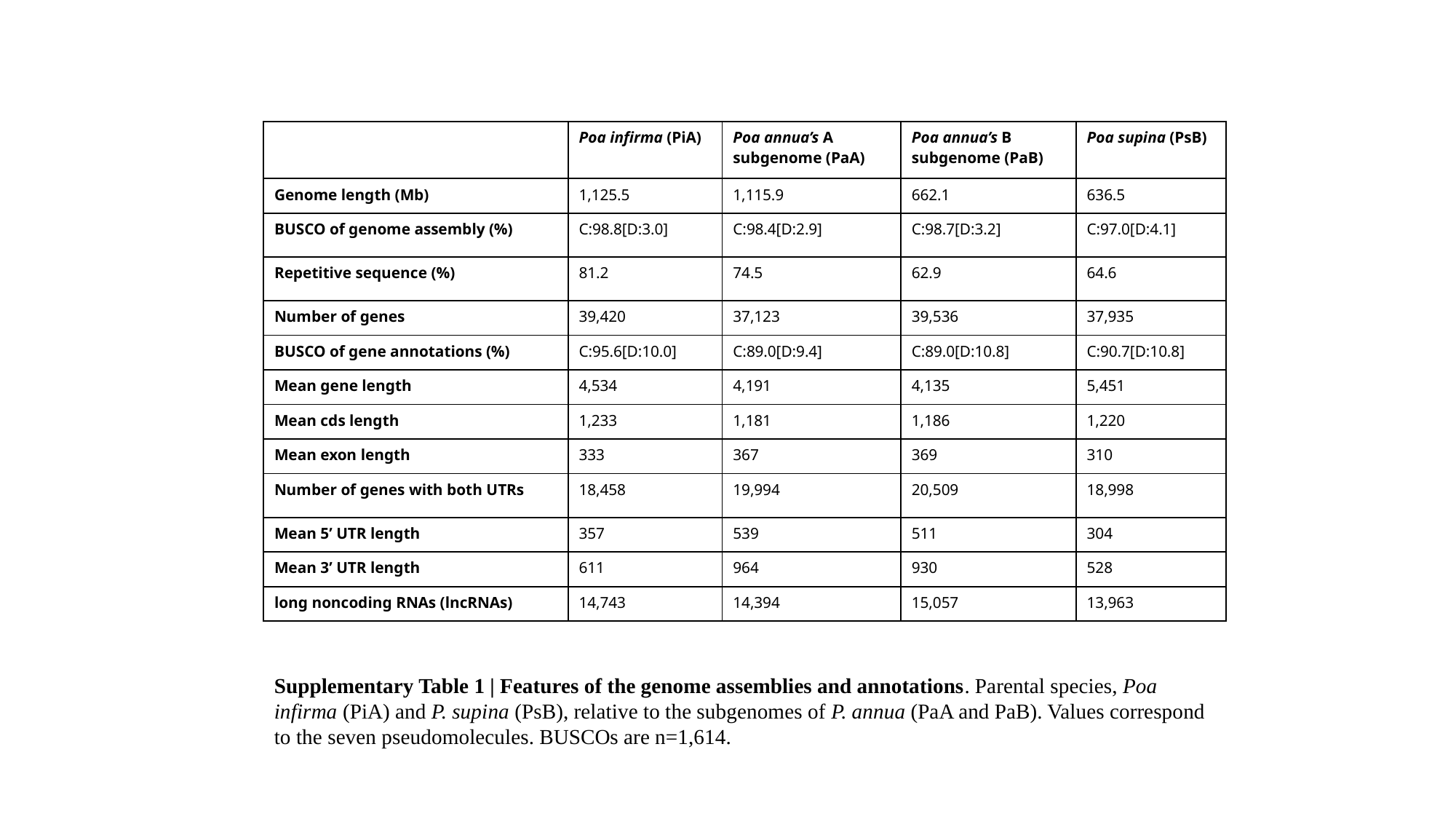

| | Poa infirma (PiA) | Poa annua’s A subgenome (PaA) | Poa annua’s B subgenome (PaB) | Poa supina (PsB) |
| --- | --- | --- | --- | --- |
| Genome length (Mb) | 1,125.5 | 1,115.9 | 662.1 | 636.5 |
| BUSCO of genome assembly (%) | C:98.8[D:3.0] | C:98.4[D:2.9] | C:98.7[D:3.2] | C:97.0[D:4.1] |
| Repetitive sequence (%) | 81.2 | 74.5 | 62.9 | 64.6 |
| Number of genes | 39,420 | 37,123 | 39,536 | 37,935 |
| BUSCO of gene annotations (%) | C:95.6[D:10.0] | C:89.0[D:9.4] | C:89.0[D:10.8] | C:90.7[D:10.8] |
| Mean gene length | 4,534 | 4,191 | 4,135 | 5,451 |
| Mean cds length | 1,233 | 1,181 | 1,186 | 1,220 |
| Mean exon length | 333 | 367 | 369 | 310 |
| Number of genes with both UTRs | 18,458 | 19,994 | 20,509 | 18,998 |
| Mean 5’ UTR length | 357 | 539 | 511 | 304 |
| Mean 3’ UTR length | 611 | 964 | 930 | 528 |
| long noncoding RNAs (lncRNAs) | 14,743 | 14,394 | 15,057 | 13,963 |
Supplementary Table 1 | Features of the genome assemblies and annotations. Parental species, Poa infirma (PiA) and P. supina (PsB), relative to the subgenomes of P. annua (PaA and PaB). Values correspond to the seven pseudomolecules. BUSCOs are n=1,614.

## Slide 2
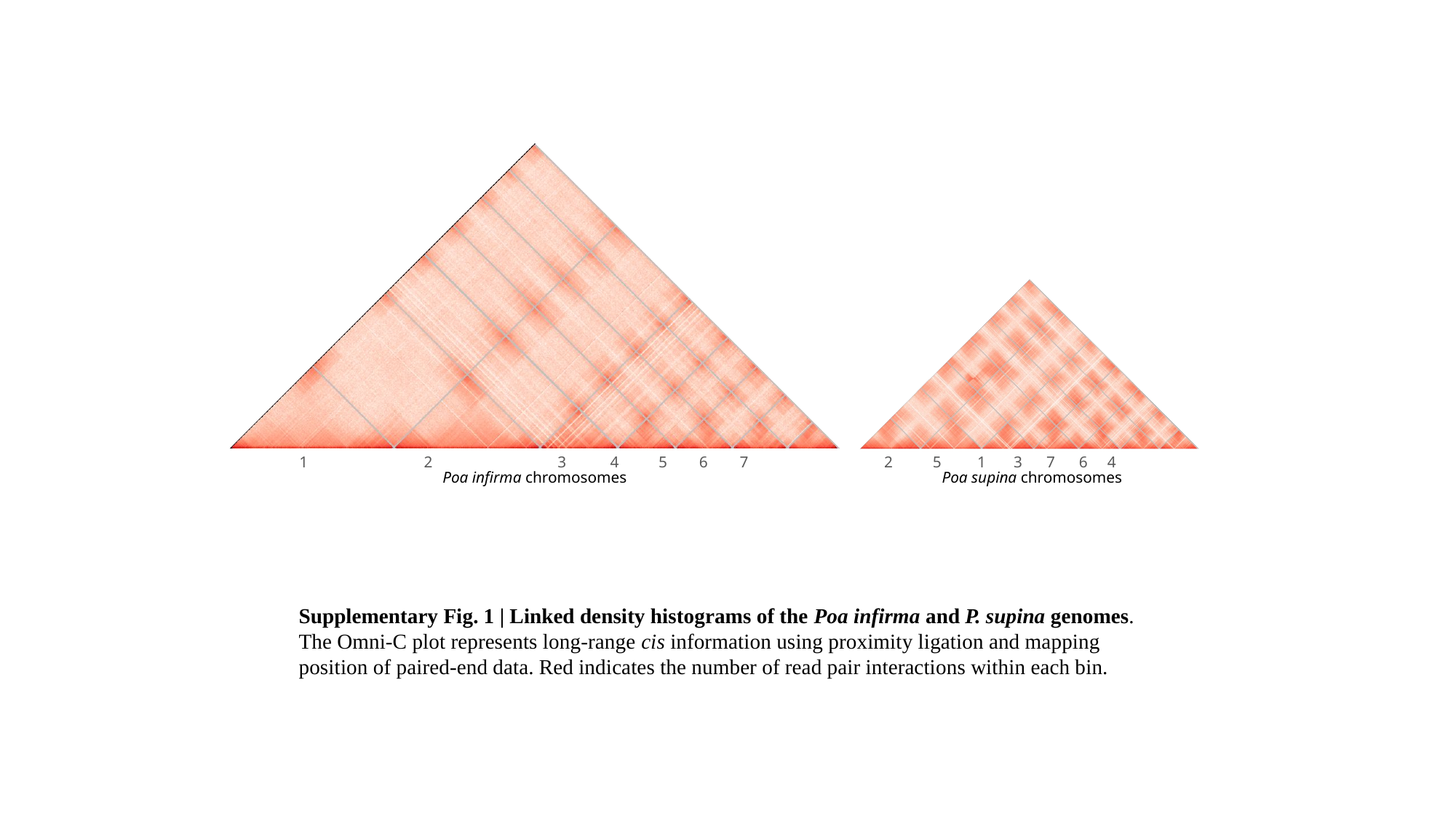

1 2	 3 4 5 6 7
2 5 1 3 7 6 4
Poa infirma chromosomes
Poa supina chromosomes
Supplementary Fig. 1 | Linked density histograms of the Poa infirma and P. supina genomes. The Omni-C plot represents long-range cis information using proximity ligation and mapping position of paired-end data. Red indicates the number of read pair interactions within each bin.

## Slide 3
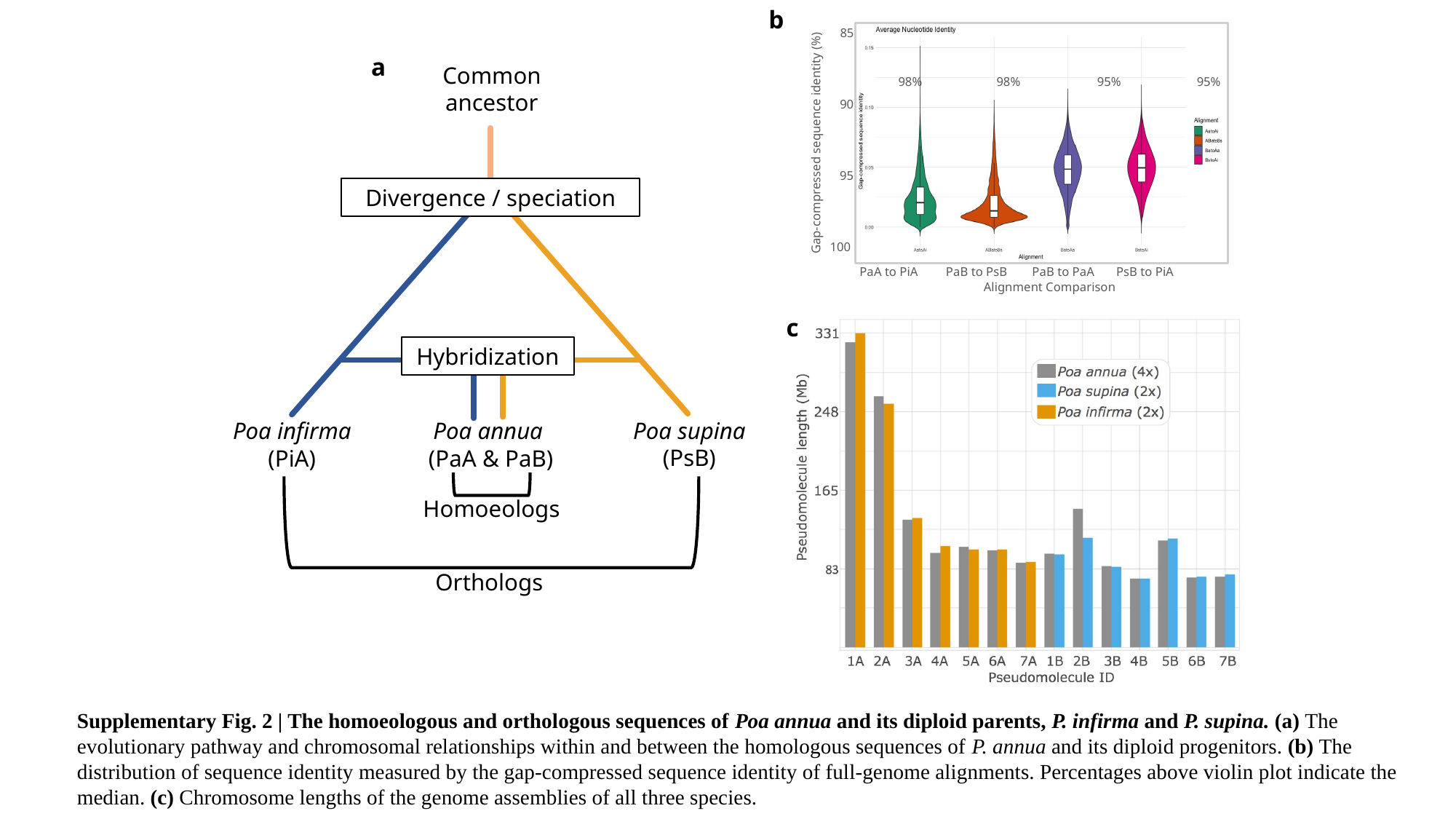

b
85
a
Common ancestor
98%
98%
95%
95%
90
Gap-compressed sequence identity (%)
95
Divergence / speciation
100
PaA to PiA PaB to PsB PaB to PaA PsB to PiA
Alignment Comparison
c
Hybridization
Poa supina (PsB)
Poa infirma (PiA)
Poa annua
 (PaA & PaB)
Homoeologs
Orthologs
Supplementary Fig. 2 | The homoeologous and orthologous sequences of Poa annua and its diploid parents, P. infirma and P. supina. (a) The evolutionary pathway and chromosomal relationships within and between the homologous sequences of P. annua and its diploid progenitors. (b) The distribution of sequence identity measured by the gap-compressed sequence identity of full-genome alignments. Percentages above violin plot indicate the median. (c) Chromosome lengths of the genome assemblies of all three species.

## Slide 4
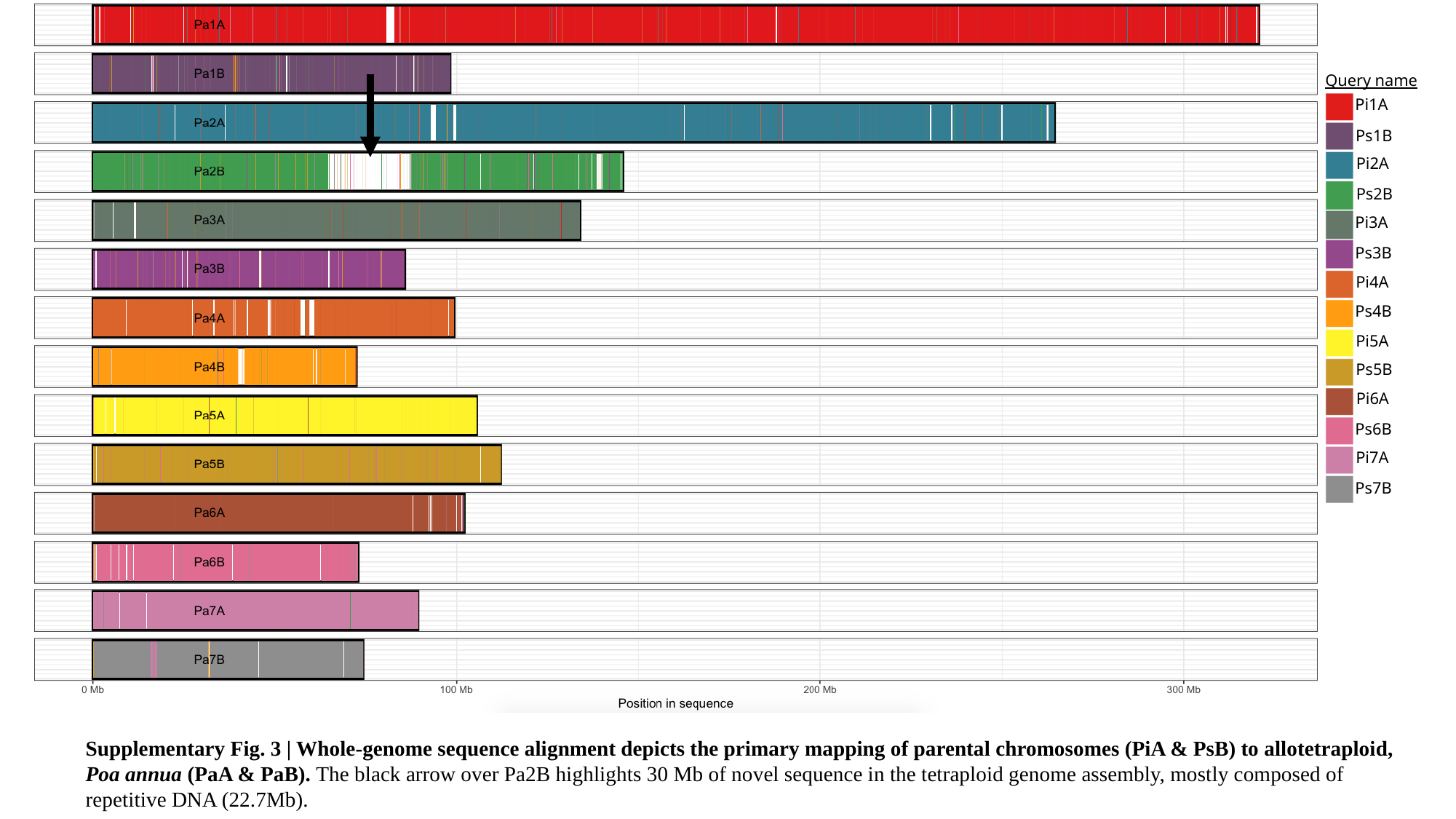

Query name
Pi1A
Ps1B
Pi2A
Ps2B
Pi3A
Ps3B
Pi4A
Ps4B
Pi5A
Ps5B
Pi6A
Ps6B
Pi7A
Ps7B
Supplementary Fig. 3 | Whole-genome sequence alignment depicts the primary mapping of parental chromosomes (PiA & PsB) to allotetraploid, Poa annua (PaA & PaB). The black arrow over Pa2B highlights 30 Mb of novel sequence in the tetraploid genome assembly, mostly composed of repetitive DNA (22.7Mb).

## Slide 5
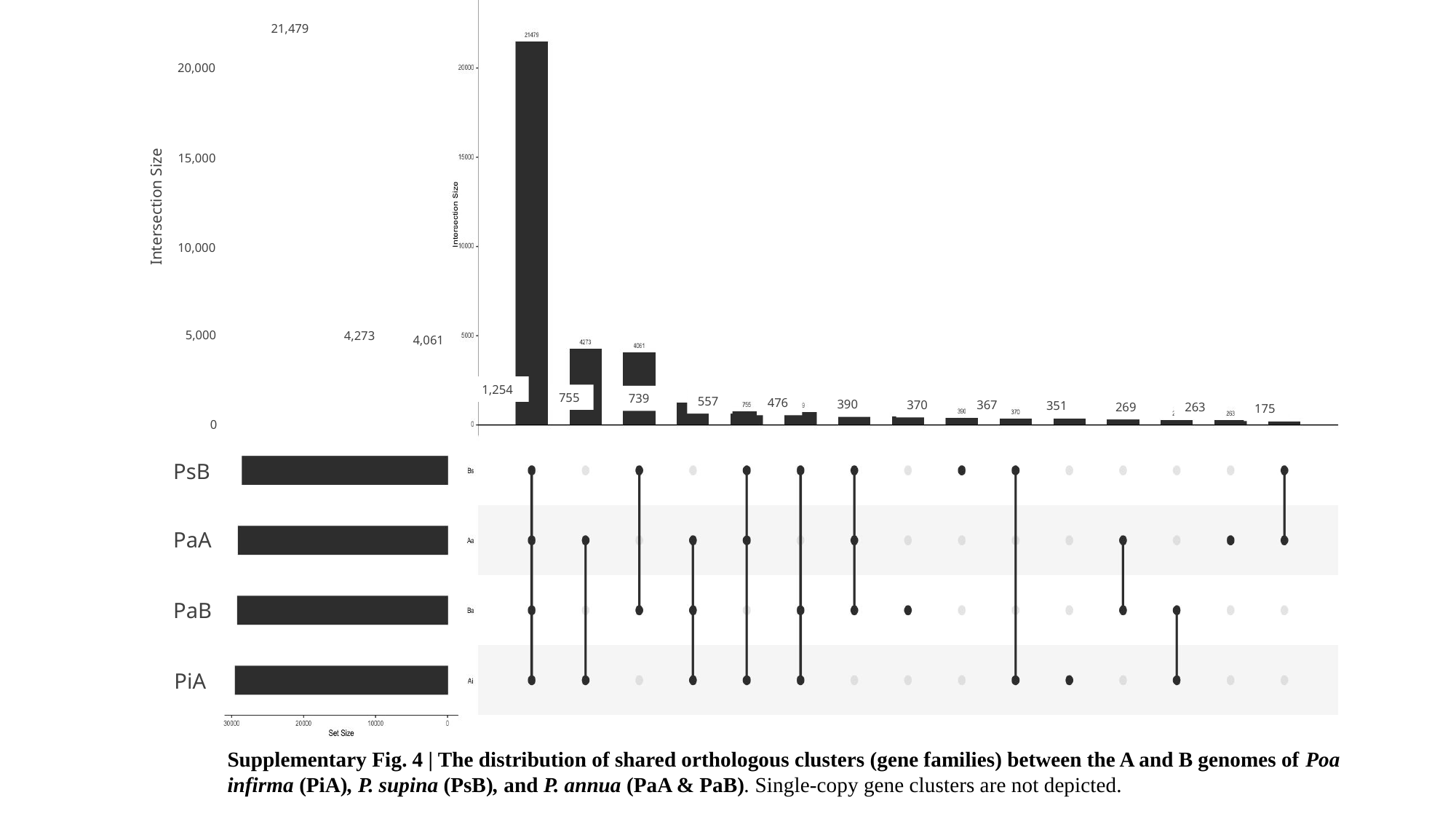

21,479
20,000
15,000
Intersection Size
10,000
5,000
4,273
4,061
1,254
755
739
557
476
390
370
367
351
269
263
175
0
PsB
PaA
PaB
PiA
Supplementary Fig. 4 | The distribution of shared orthologous clusters (gene families) between the A and B genomes of Poa infirma (PiA), P. supina (PsB), and P. annua (PaA & PaB). Single-copy gene clusters are not depicted.

## Slide 6
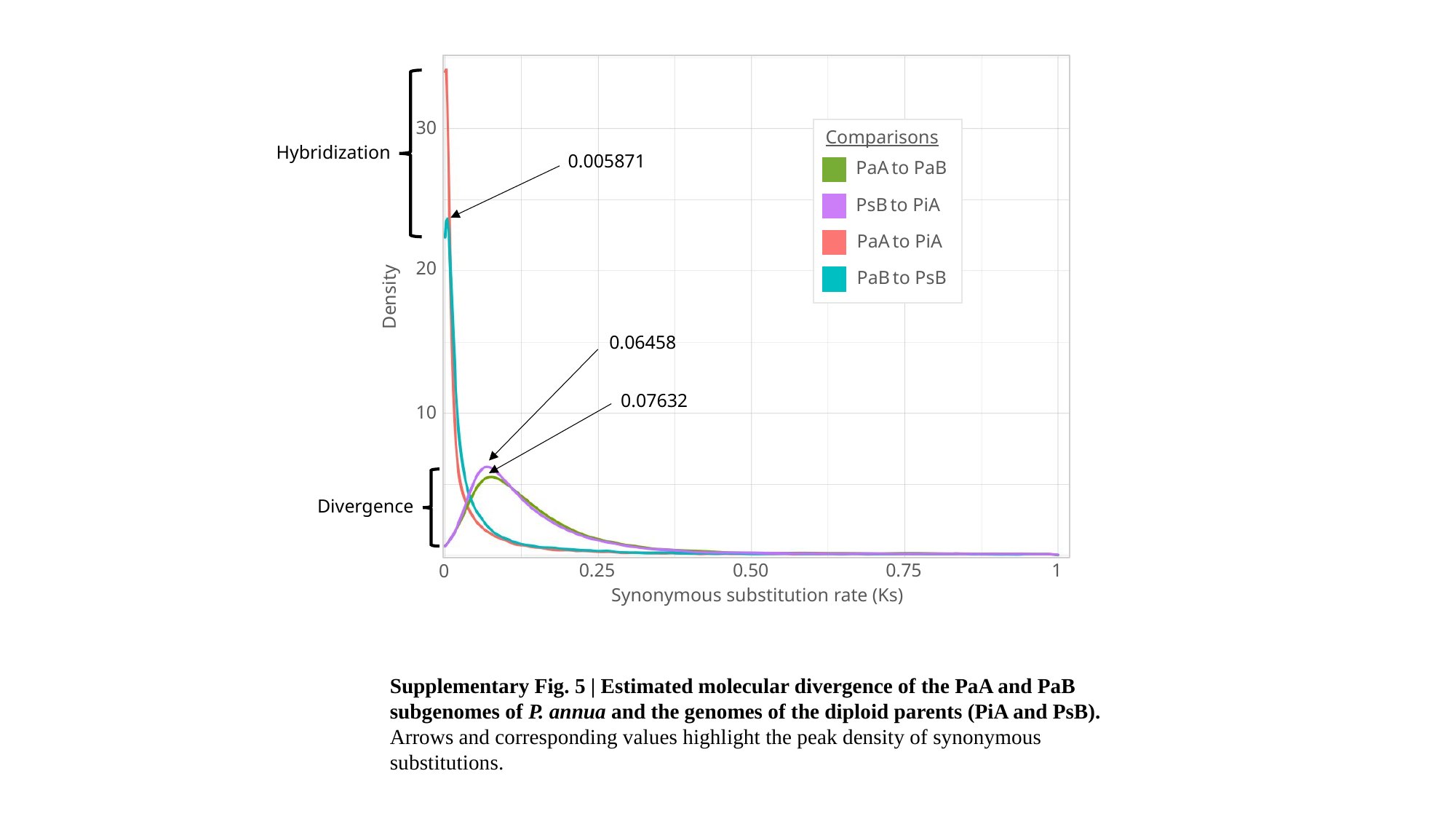

30
Comparisons
Hybridization
0.005871
PaA to PaB
PsB to PiA
PaA to PiA
20
PaB to PsB
Density
0.06458
0.07632
10
Divergence
0.50
0.25
1
0.75
0
Synonymous substitution rate (Ks)
Supplementary Fig. 5 | Estimated molecular divergence of the PaA and PaB subgenomes of P. annua and the genomes of the diploid parents (PiA and PsB). Arrows and corresponding values highlight the peak density of synonymous substitutions.

## Slide 7
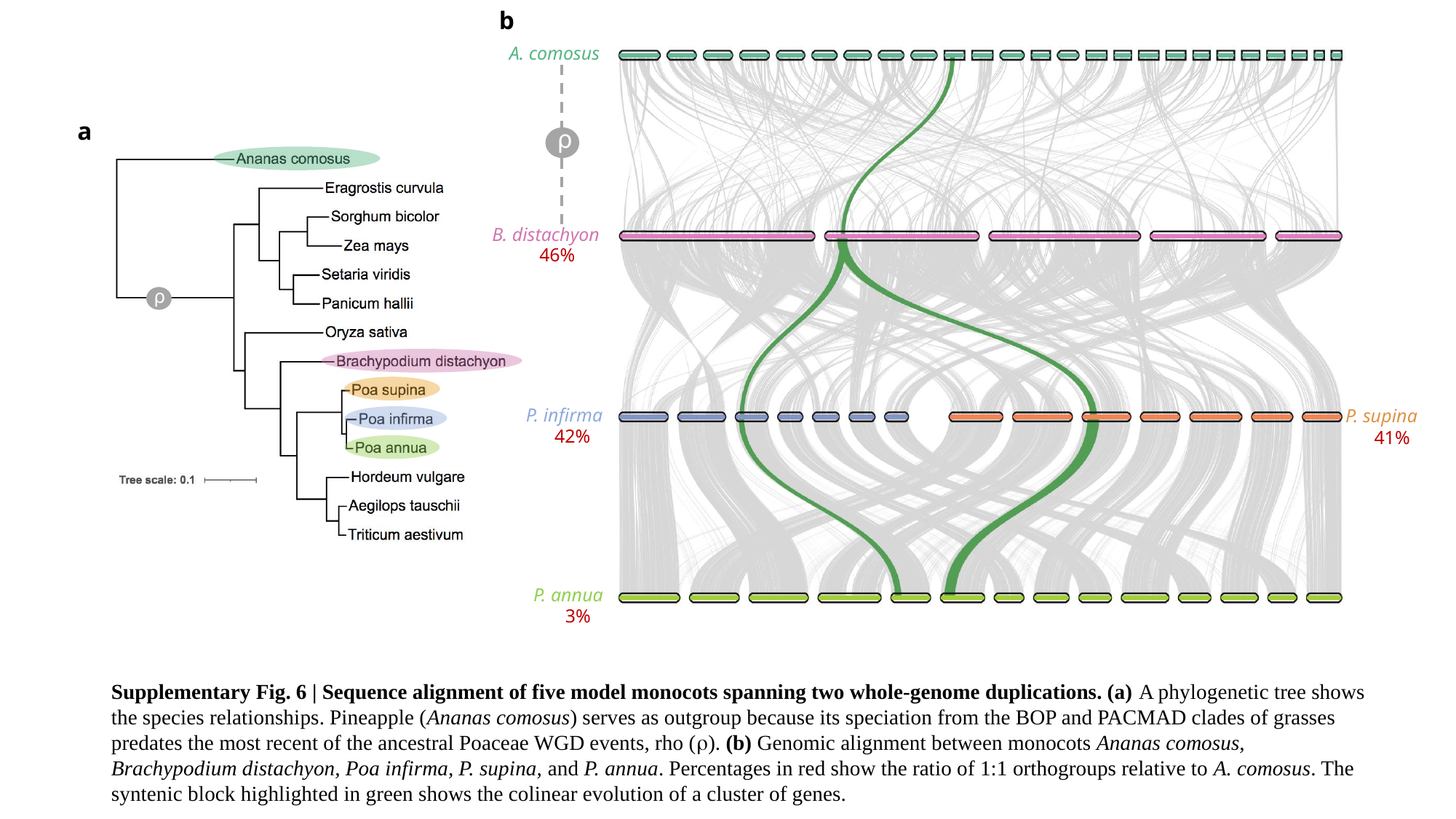

b
A. comosus
a
⍴
B. distachyon
46%
⍴
P. infirma
P. supina
42%
41%
P. annua
3%
Supplementary Fig. 6 | Sequence alignment of five model monocots spanning two whole-genome duplications. (a) A phylogenetic tree shows the species relationships. Pineapple (Ananas comosus) serves as outgroup because its speciation from the BOP and PACMAD clades of grasses predates the most recent of the ancestral Poaceae WGD events, rho (ρ). (b) Genomic alignment between monocots Ananas comosus, Brachypodium distachyon, Poa infirma, P. supina, and P. annua. Percentages in red show the ratio of 1:1 orthogroups relative to A. comosus. The syntenic block highlighted in green shows the colinear evolution of a cluster of genes.

## Slide 8
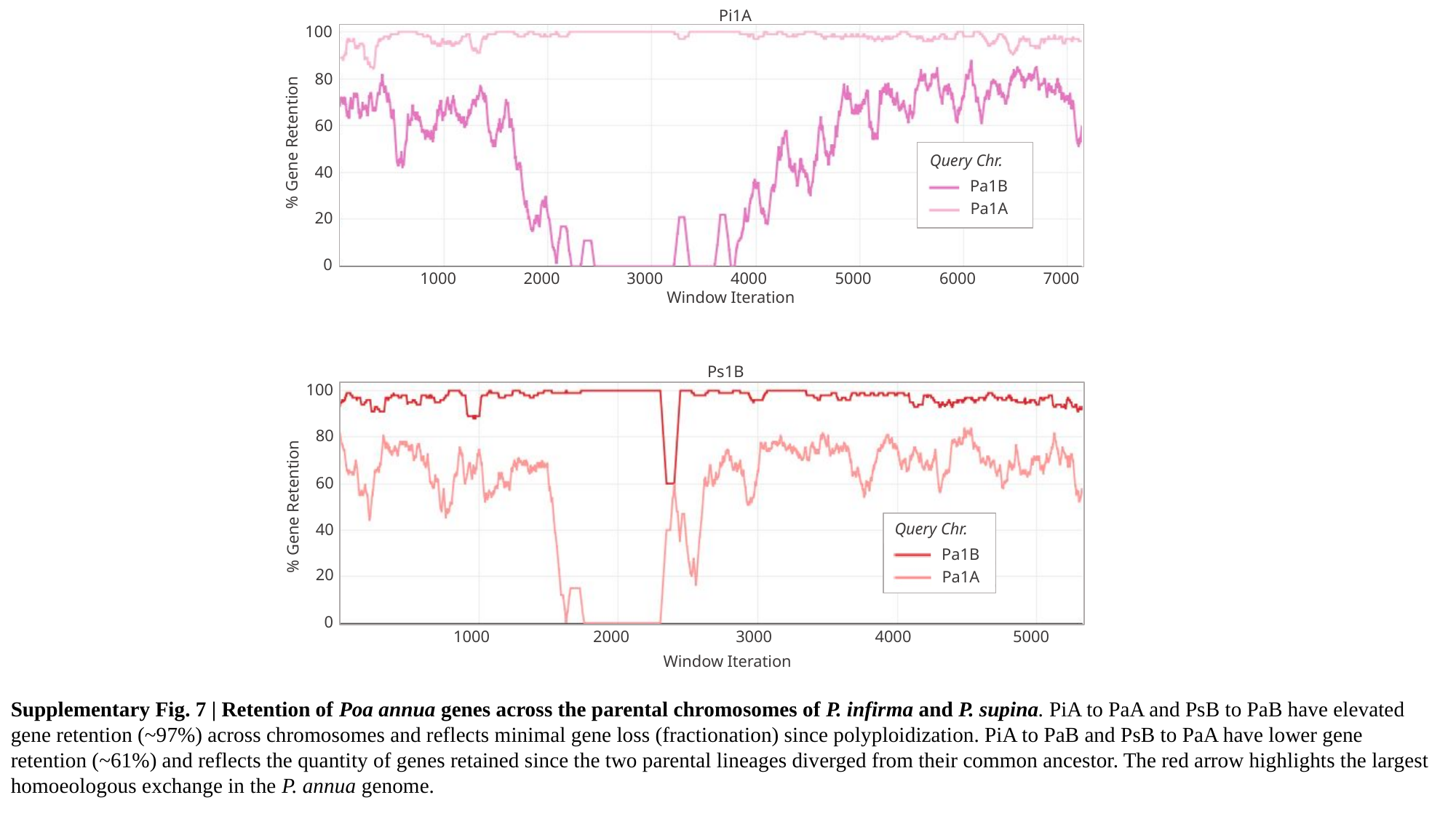

Pi1A
100
80
60
40
20
0
% Gene Retention
Query Chr.
Pa1B
Pa1A
5000
4000
3000
2000
1000
6000
7000
Window Iteration
Ps1B
100
80
60
40
20
0
% Gene Retention
Query Chr.
Pa1B
Pa1A
5000
4000
3000
2000
1000
Window Iteration
Supplementary Fig. 7 | Retention of Poa annua genes across the parental chromosomes of P. infirma and P. supina. PiA to PaA and PsB to PaB have elevated gene retention (~97%) across chromosomes and reflects minimal gene loss (fractionation) since polyploidization. PiA to PaB and PsB to PaA have lower gene retention (~61%) and reflects the quantity of genes retained since the two parental lineages diverged from their common ancestor. The red arrow highlights the largest homoeologous exchange in the P. annua genome.

## Slide 9
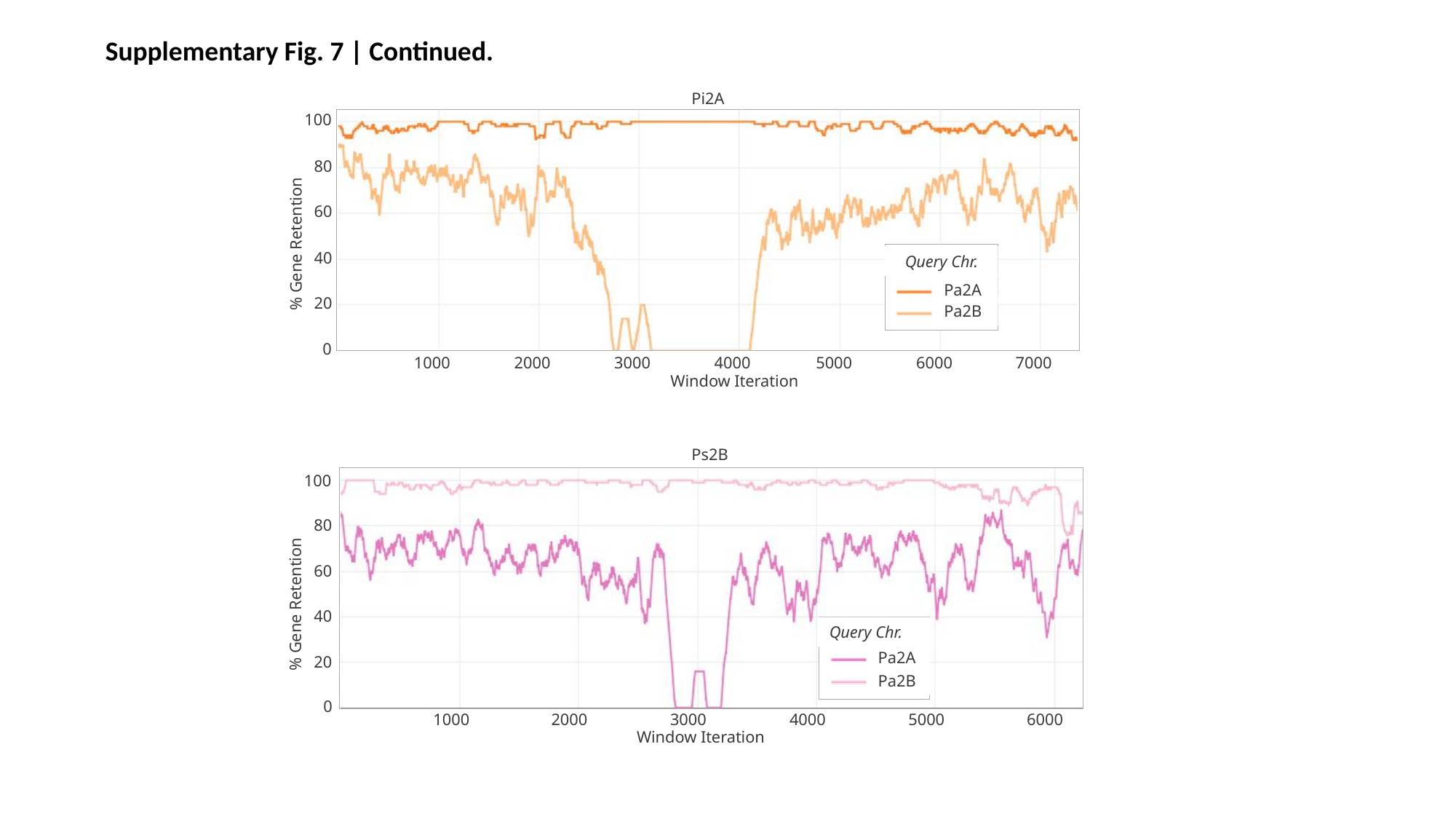

Supplementary Fig. 7 | Continued.
Pi2A
100
80
60
40
20
0
% Gene Retention
Query Chr.
Pa2A
Pa2B
7000
6000
5000
4000
3000
2000
1000
Window Iteration
Ps2B
100
80
60
40
20
0
% Gene Retention
Query Chr.
Pa2A
Pa2B
6000
5000
4000
3000
2000
1000
Window Iteration

## Slide 10
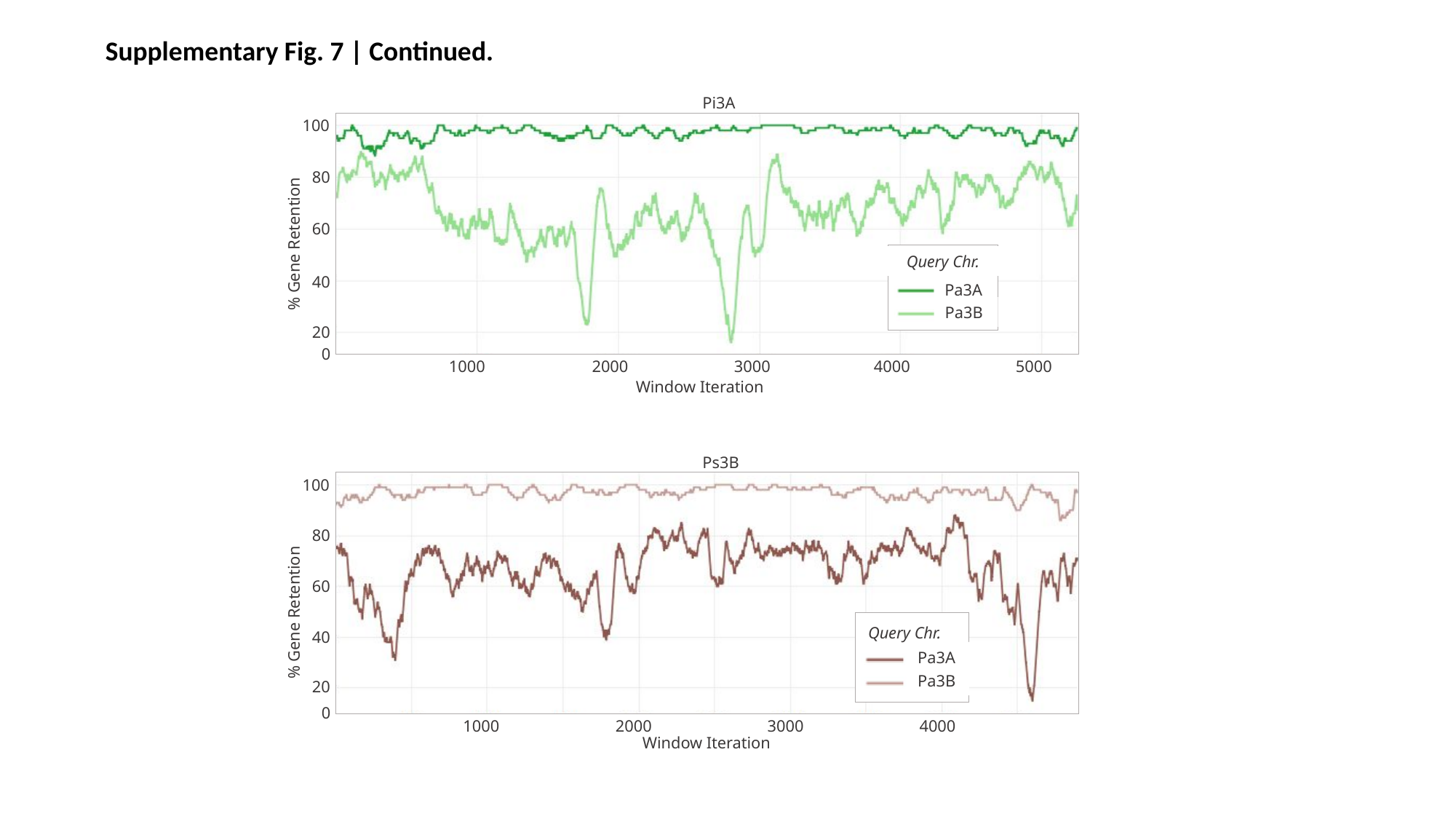

Supplementary Fig. 7 | Continued.
Pi3A
100
80
60
40
20
0
% Gene Retention
Query Chr.
Pa3A
Pa3B
5000
4000
3000
2000
1000
Window Iteration
Ps3B
100
80
60
40
20
0
% Gene Retention
Query Chr.
Pa3A
Pa3B
4000
3000
2000
1000
Window Iteration

## Slide 11
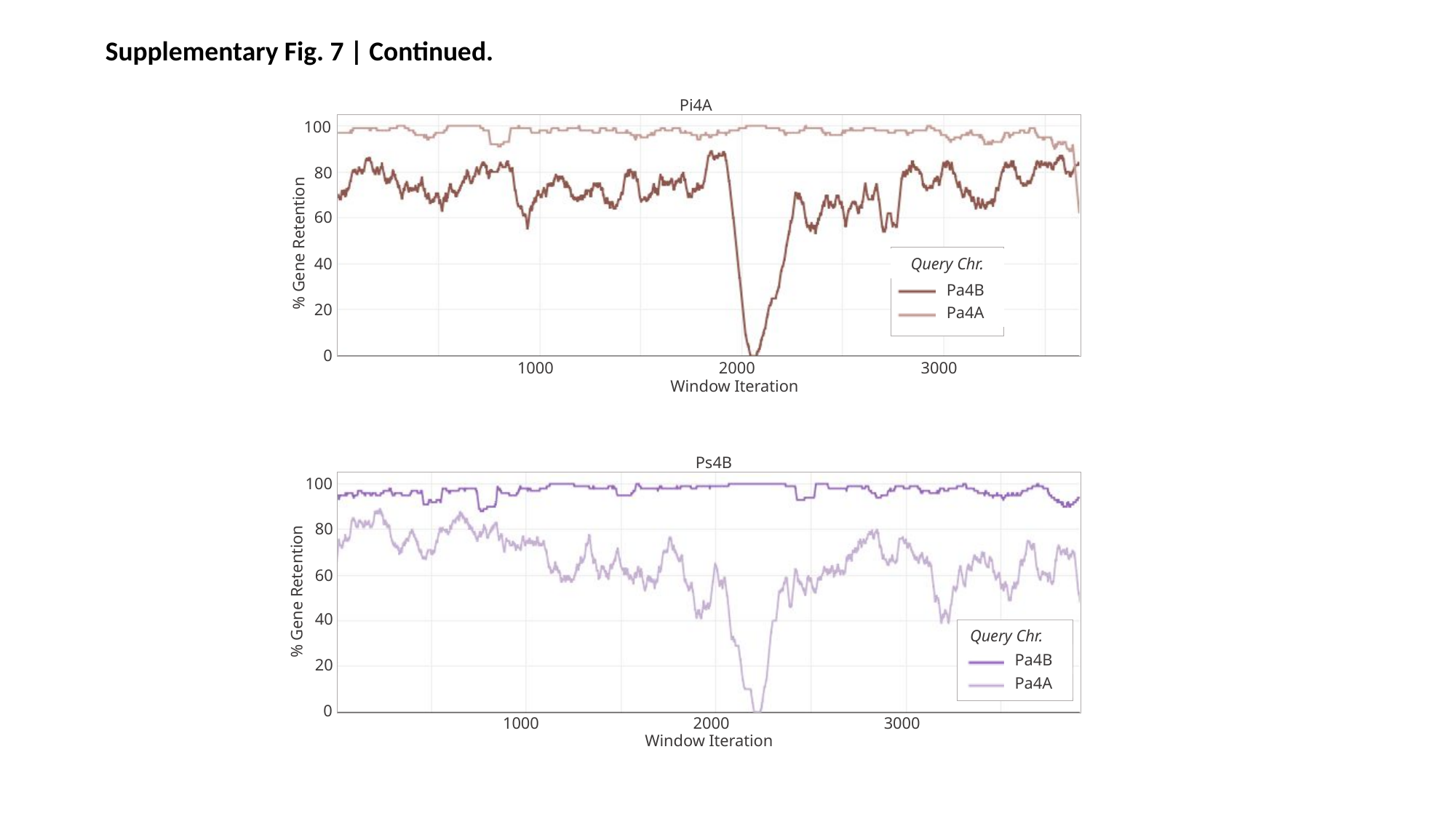

Supplementary Fig. 7 | Continued.
Pi4A
100
80
60
40
20
0
% Gene Retention
Query Chr.
Pa4B
Pa4A
3000
2000
1000
Window Iteration
Ps4B
100
80
60
40
20
0
% Gene Retention
Query Chr.
Pa4B
Pa4A
3000
2000
1000
Window Iteration

## Slide 12
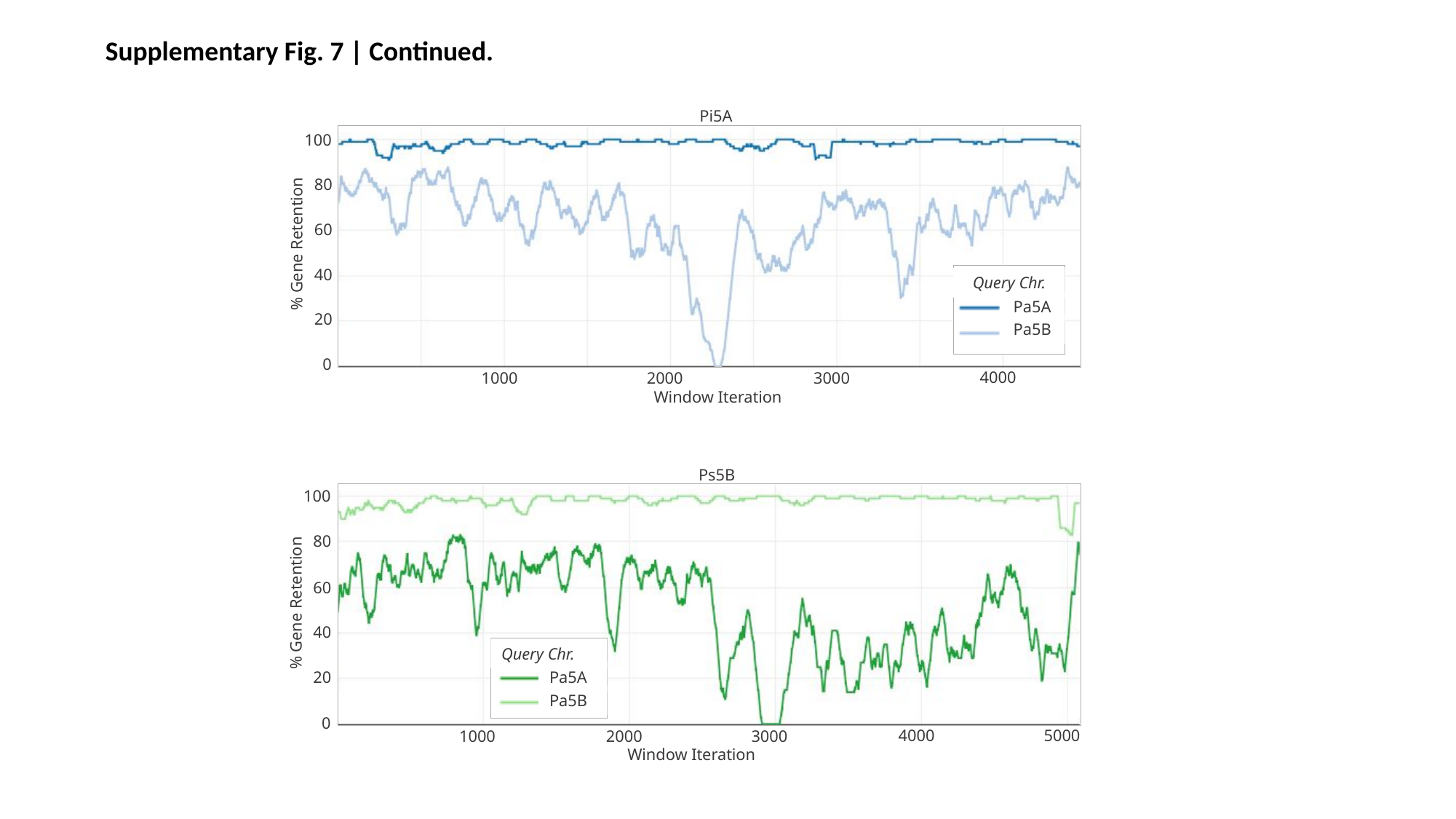

Supplementary Fig. 7 | Continued.
Pi5A
100
80
60
40
20
0
% Gene Retention
Query Chr.
Pa5A
Pa5B
3000
2000
1000
Window Iteration
4000
Ps5B
100
80
60
40
20
0
% Gene Retention
Query Chr.
Pa5A
Pa5B
3000
2000
1000
Window Iteration
5000
4000

## Slide 13
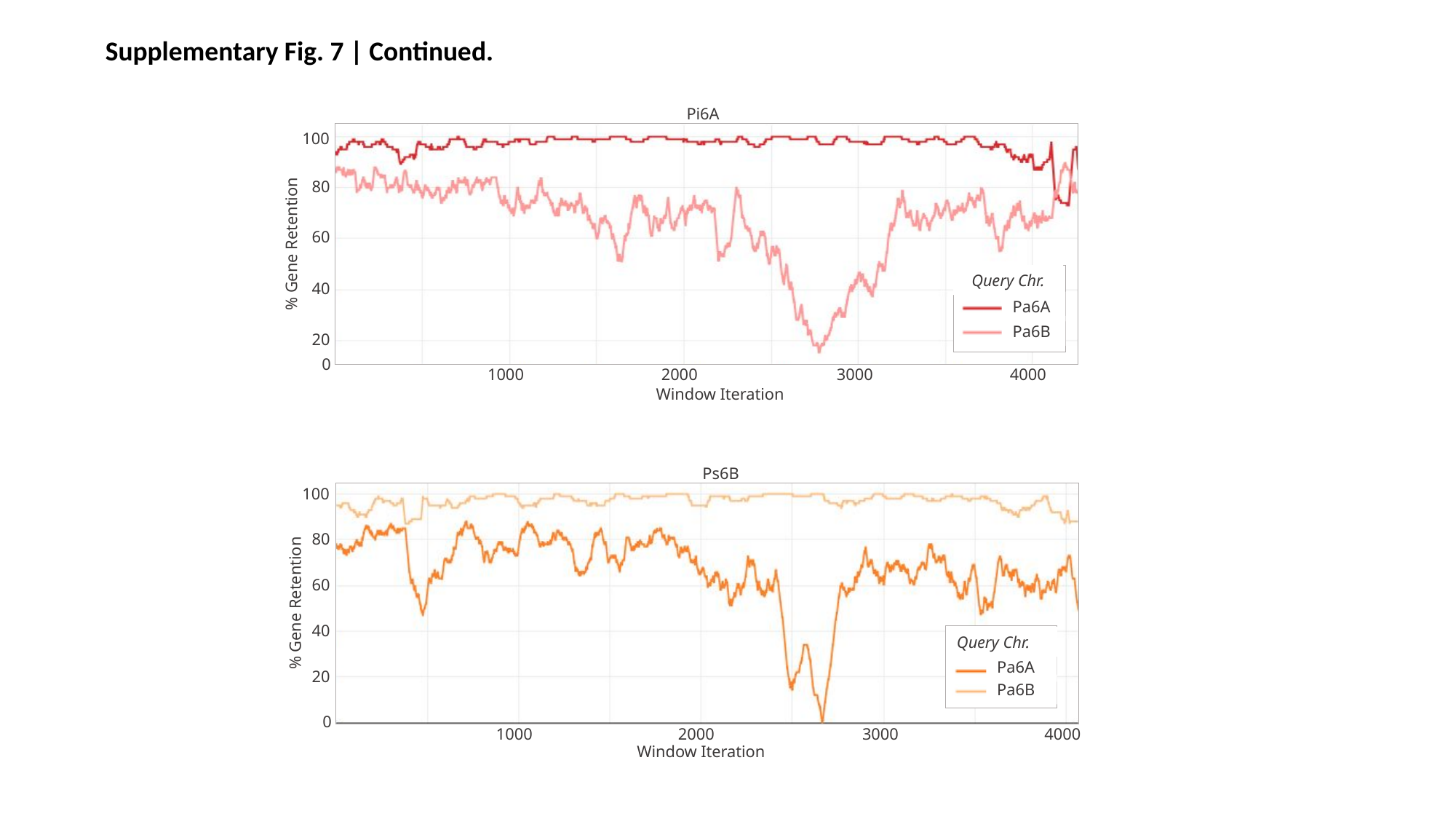

Supplementary Fig. 7 | Continued.
Pi6A
100
80
60
40
20
0
% Gene Retention
Query Chr.
Pa6A
Pa6B
3000
2000
1000
Window Iteration
4000
Ps6B
100
80
60
40
20
0
% Gene Retention
Query Chr.
Pa6A
Pa6B
3000
2000
1000
Window Iteration
4000

## Slide 14
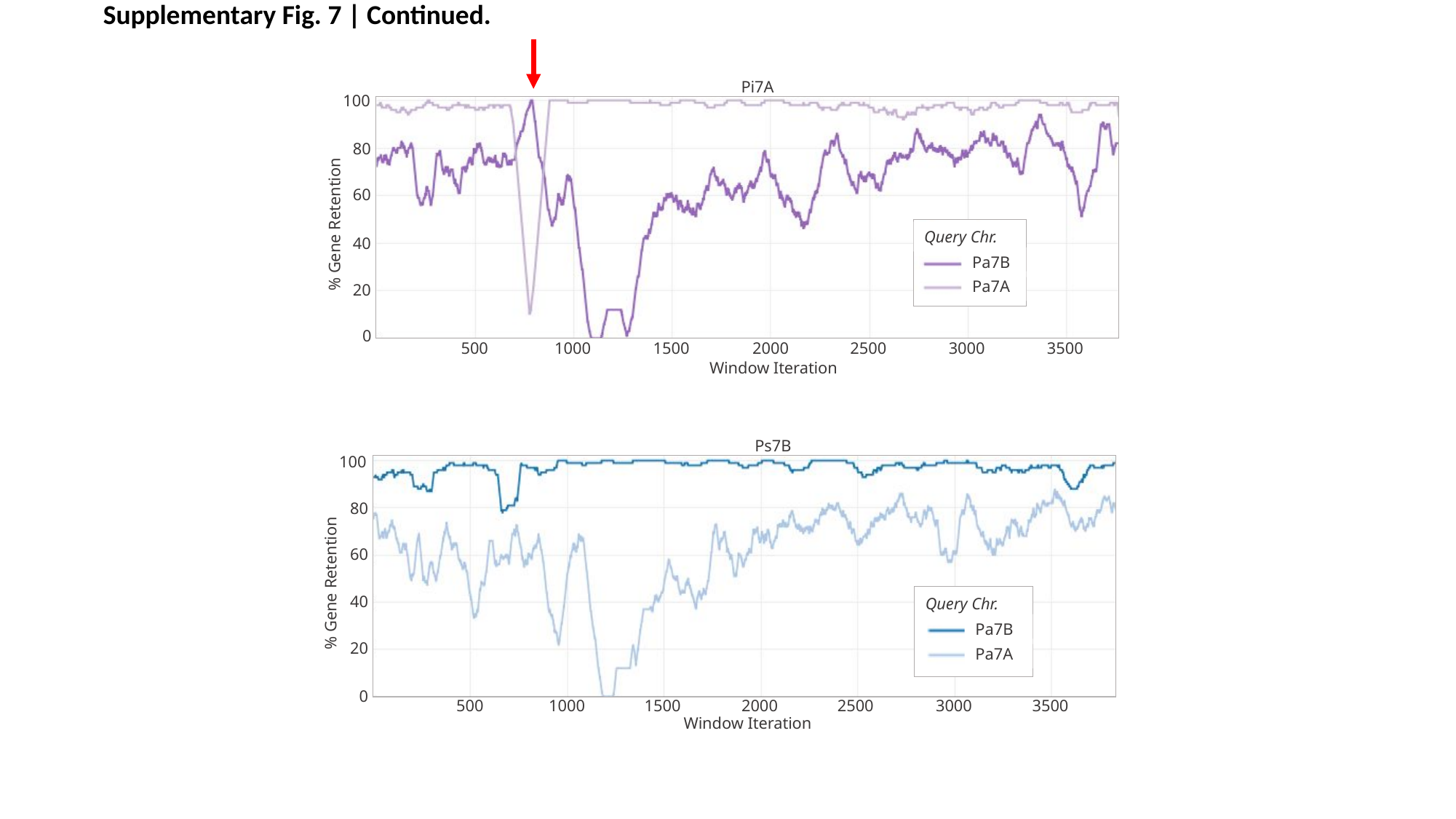

Supplementary Fig. 7 | Continued.
Pi7A
100
80
60
40
20
0
% Gene Retention
Query Chr.
Pa7B
Pa7A
3500
3000
2500
2000
1500
1000
500
Window Iteration
Ps7B
100
80
60
40
20
0
% Gene Retention
Query Chr.
Pa7B
Pa7A
3500
3000
2500
2000
1500
1000
500
Window Iteration

## Slide 15
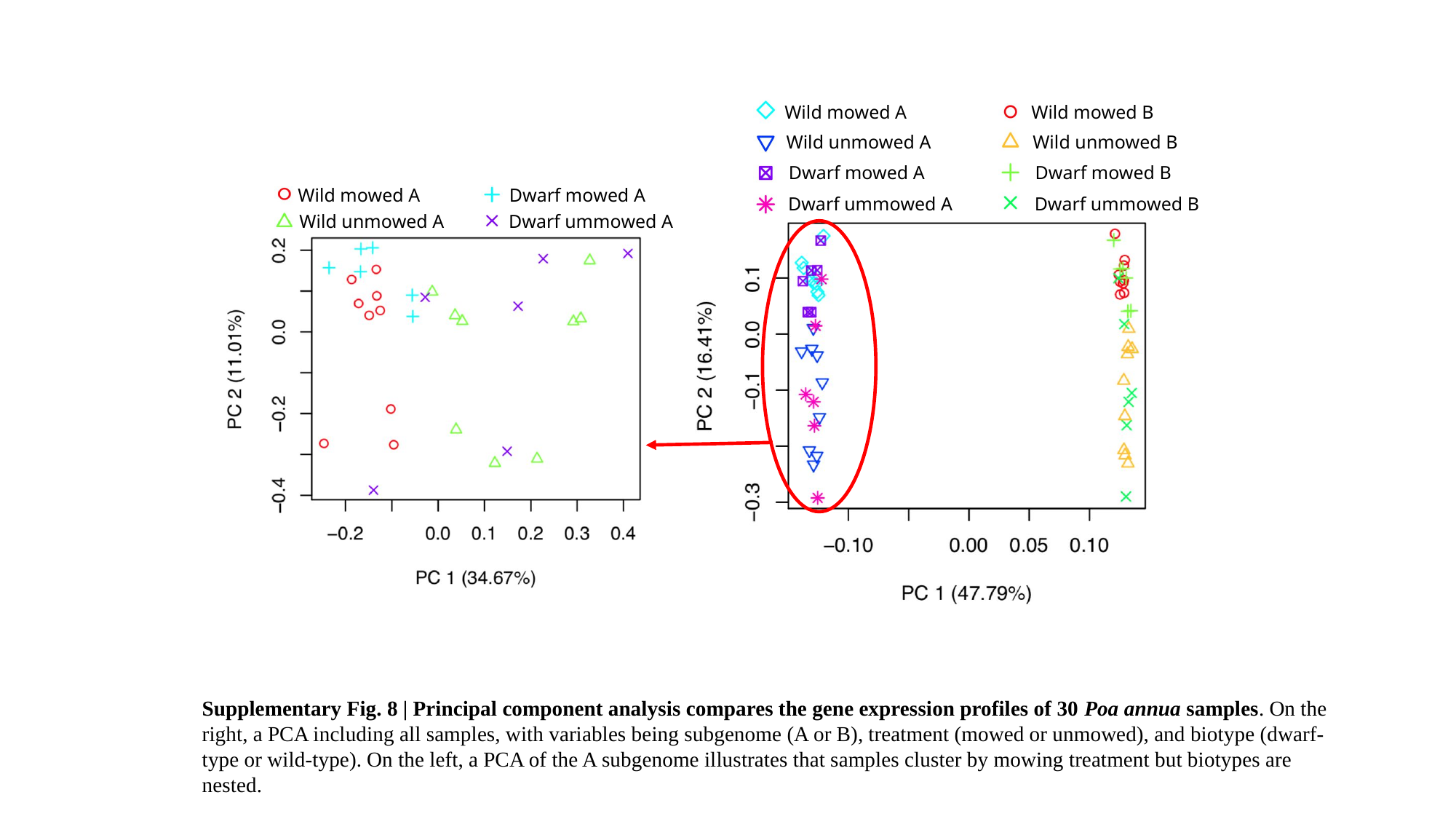

Wild mowed A
Wild mowed B
Wild unmowed A
Wild unmowed B
Dwarf mowed A
Dwarf mowed B
Wild mowed A
Dwarf mowed A
Dwarf ummowed A
Dwarf ummowed B
Wild unmowed A
Dwarf ummowed A
Supplementary Fig. 8 | Principal component analysis compares the gene expression profiles of 30 Poa annua samples. On the right, a PCA including all samples, with variables being subgenome (A or B), treatment (mowed or unmowed), and biotype (dwarf-type or wild-type). On the left, a PCA of the A subgenome illustrates that samples cluster by mowing treatment but biotypes are nested.

## Slide 16
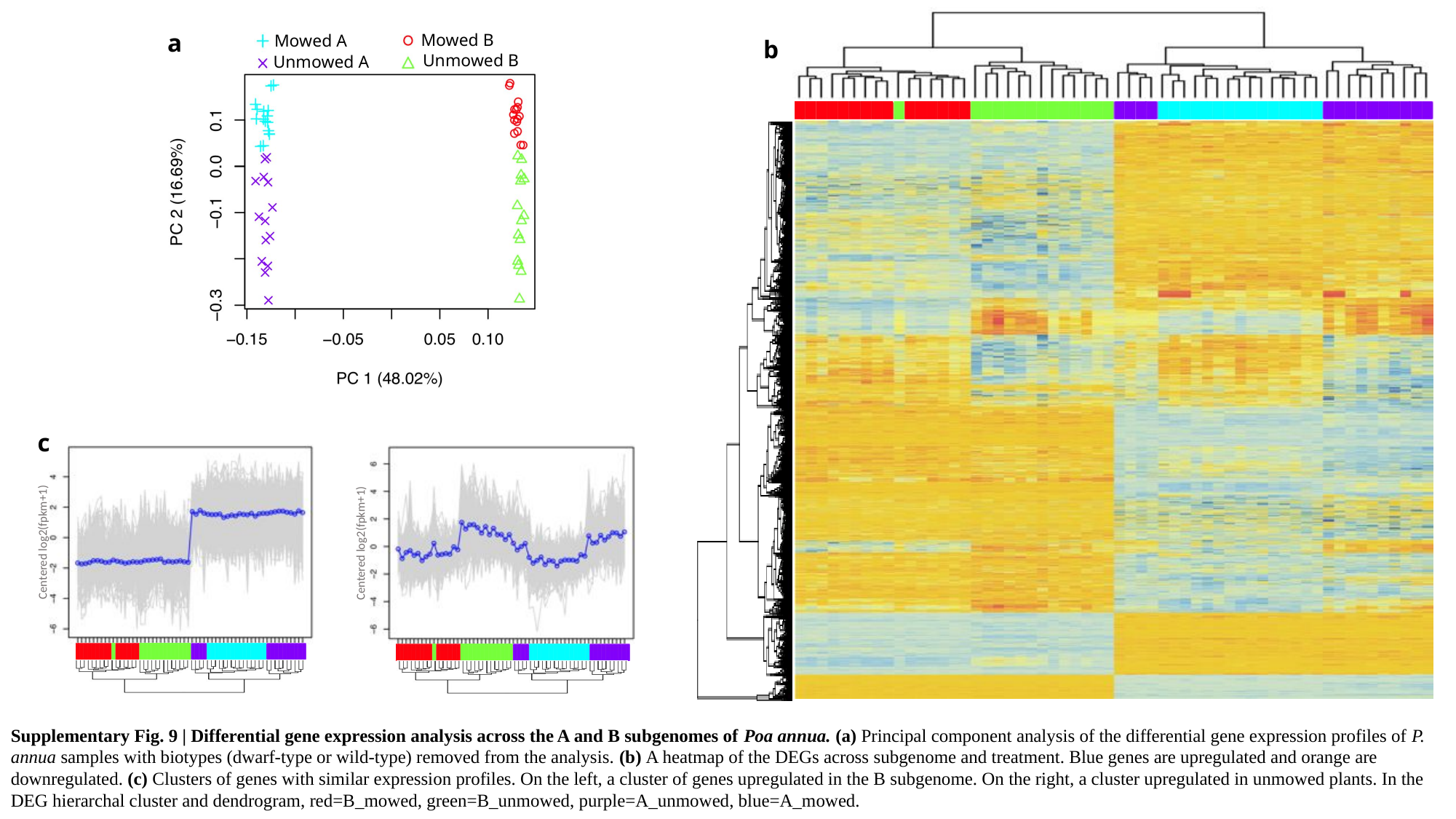

a
Mowed B
Mowed A
Unmowed B
Unmowed A
b
c
Centered log2(fpkm+1)
Centered log2(fpkm+1)
Supplementary Fig. 9 | Differential gene expression analysis across the A and B subgenomes of Poa annua. (a) Principal component analysis of the differential gene expression profiles of P. annua samples with biotypes (dwarf-type or wild-type) removed from the analysis. (b) A heatmap of the DEGs across subgenome and treatment. Blue genes are upregulated and orange are downregulated. (c) Clusters of genes with similar expression profiles. On the left, a cluster of genes upregulated in the B subgenome. On the right, a cluster upregulated in unmowed plants. In the DEG hierarchal cluster and dendrogram, red=B_mowed, green=B_unmowed, purple=A_unmowed, blue=A_mowed.

## Slide 17
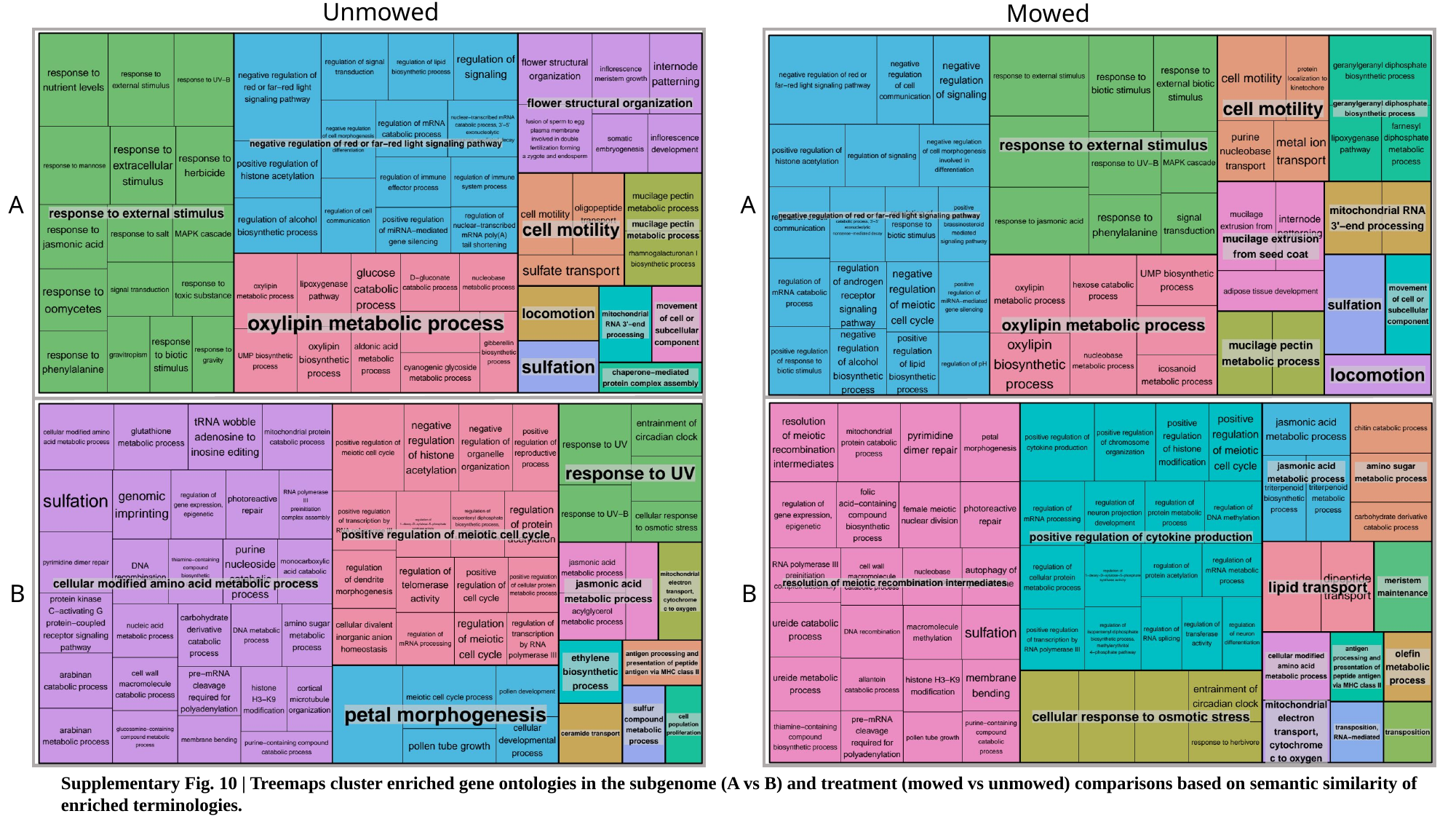

Unmowed
Mowed
A
A
B
B
Supplementary Fig. 10 | Treemaps cluster enriched gene ontologies in the subgenome (A vs B) and treatment (mowed vs unmowed) comparisons based on semantic similarity of enriched terminologies.

## Slide 18
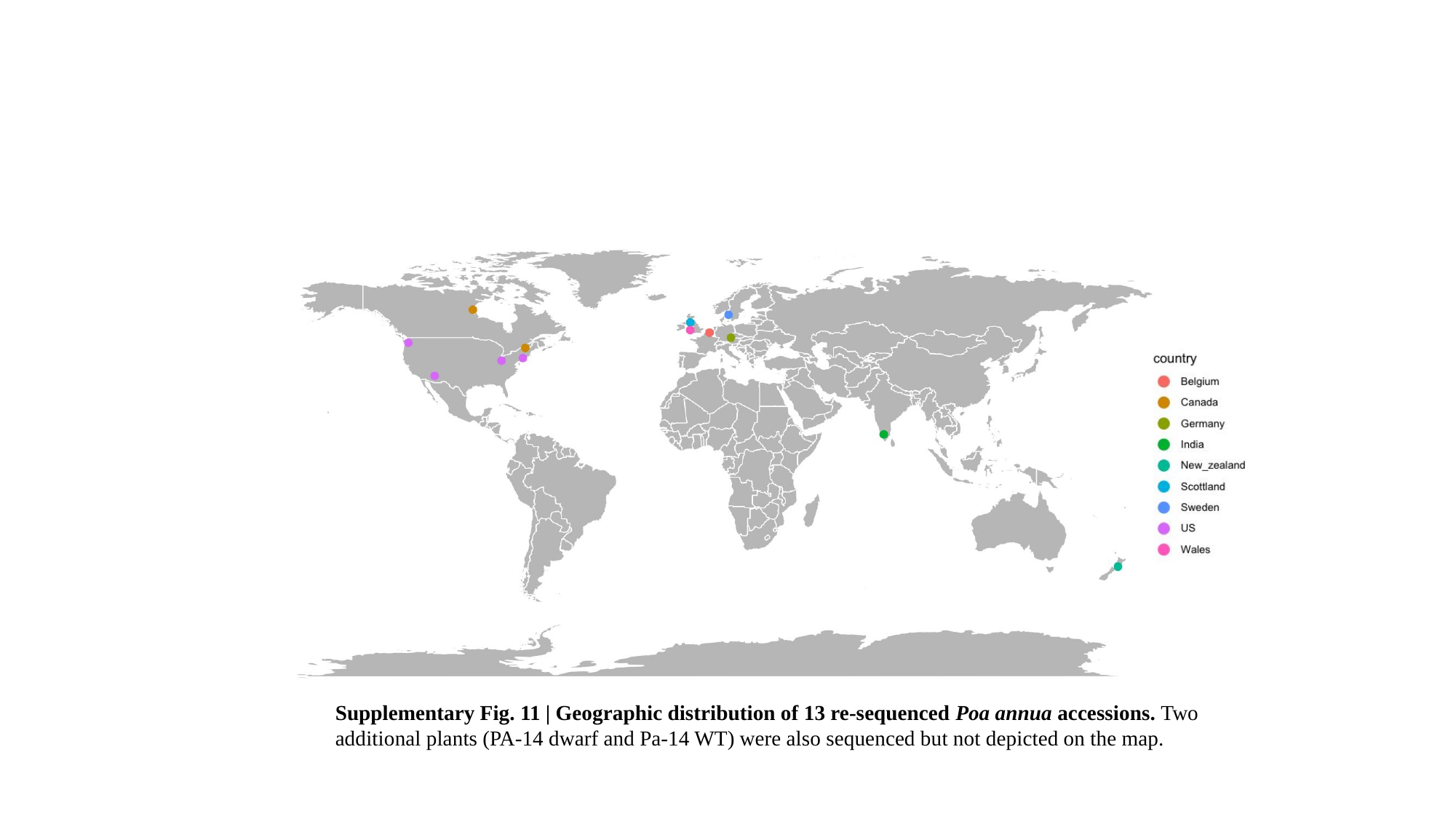

Supplementary Fig. 11 | Geographic distribution of 13 re-sequenced Poa annua accessions. Two additional plants (PA-14 dwarf and Pa-14 WT) were also sequenced but not depicted on the map.

## Slide 19
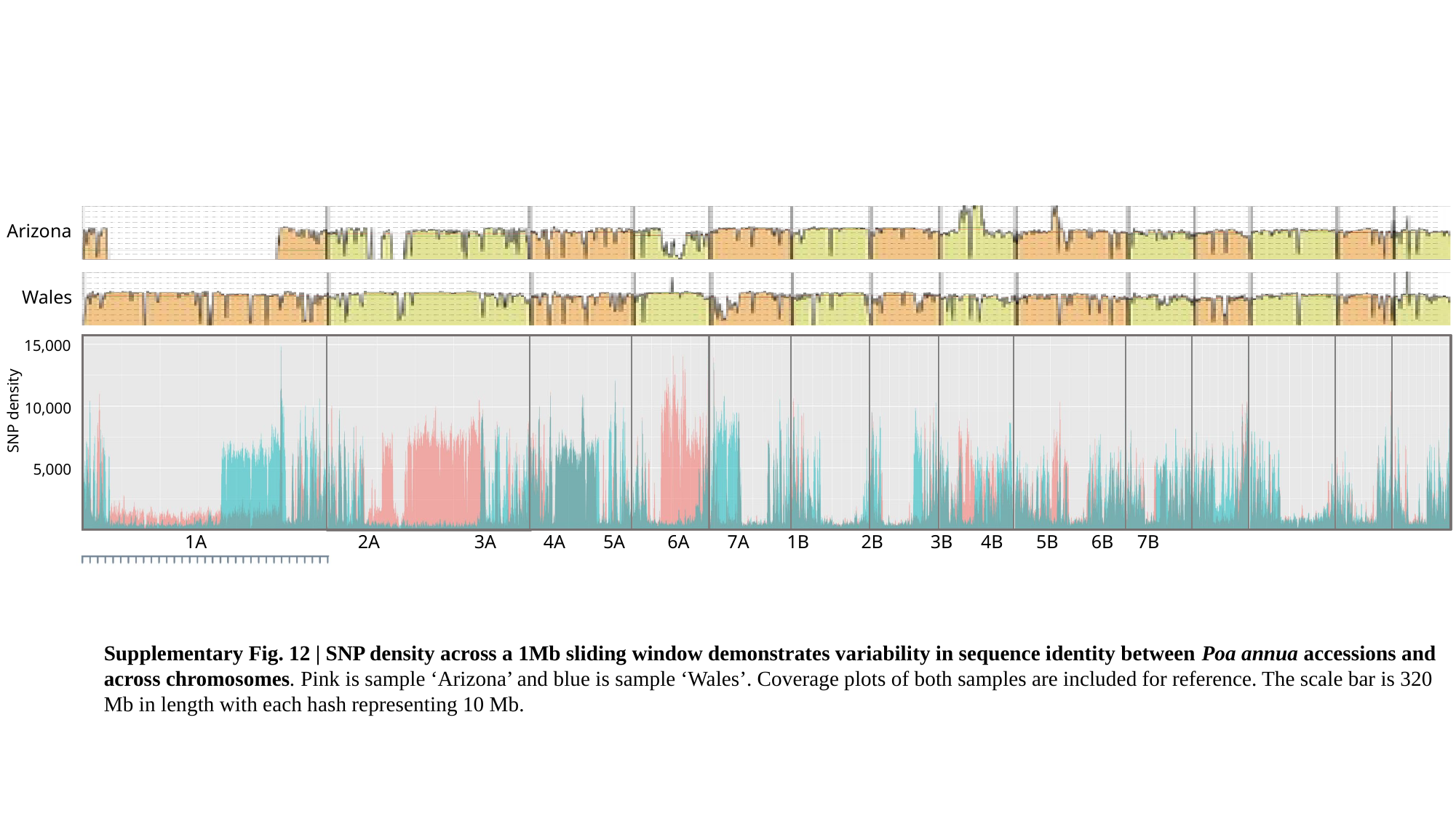

Arizona
Wales
15,000
SNP density
10,000
5,000
1A 2A 3A 4A 5A 6A 7A 1B 2B 3B 4B 5B 6B 7B
Supplementary Fig. 12 | SNP density across a 1Mb sliding window demonstrates variability in sequence identity between Poa annua accessions and across chromosomes. Pink is sample ‘Arizona’ and blue is sample ‘Wales’. Coverage plots of both samples are included for reference. The scale bar is 320 Mb in length with each hash representing 10 Mb.

## Slide 20
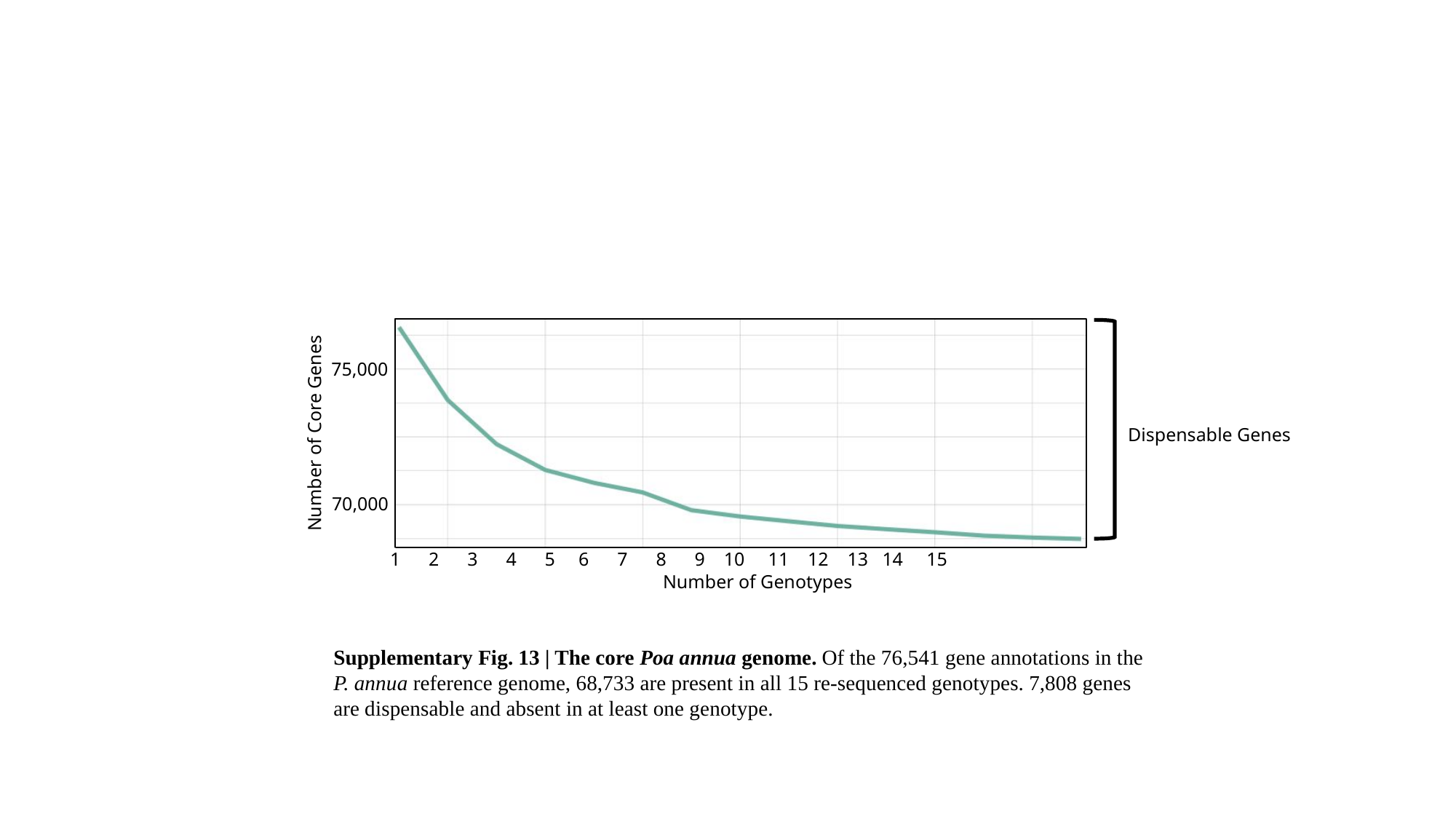

75,000
Number of Core Genes
Dispensable Genes
70,000
1 2 3 4 5 6 7 8 9 10 11 12 13 14 15
Number of Genotypes
Supplementary Fig. 13 | The core Poa annua genome. Of the 76,541 gene annotations in the P. annua reference genome, 68,733 are present in all 15 re-sequenced genotypes. 7,808 genes are dispensable and absent in at least one genotype.

## Slide 21
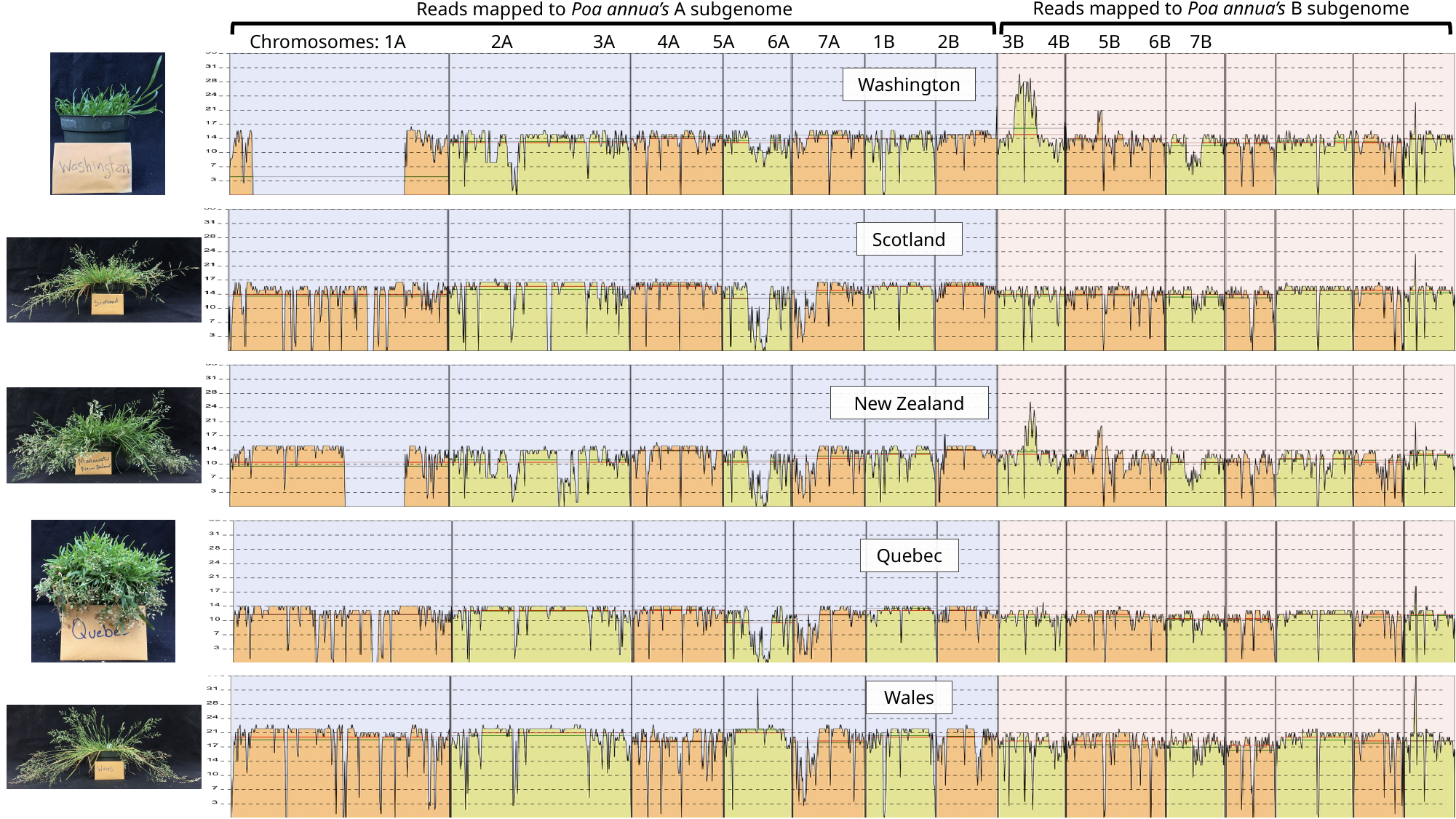

Reads mapped to Poa annua’s B subgenome
Reads mapped to Poa annua’s A subgenome
Chromosomes: 1A 2A 3A 4A 5A 6A 7A 1B 2B 3B 4B 5B 6B 7B
Supplementary Fig. 14.
Washington
Scotland
New Zealand
Quebec
Wales

## Slide 22
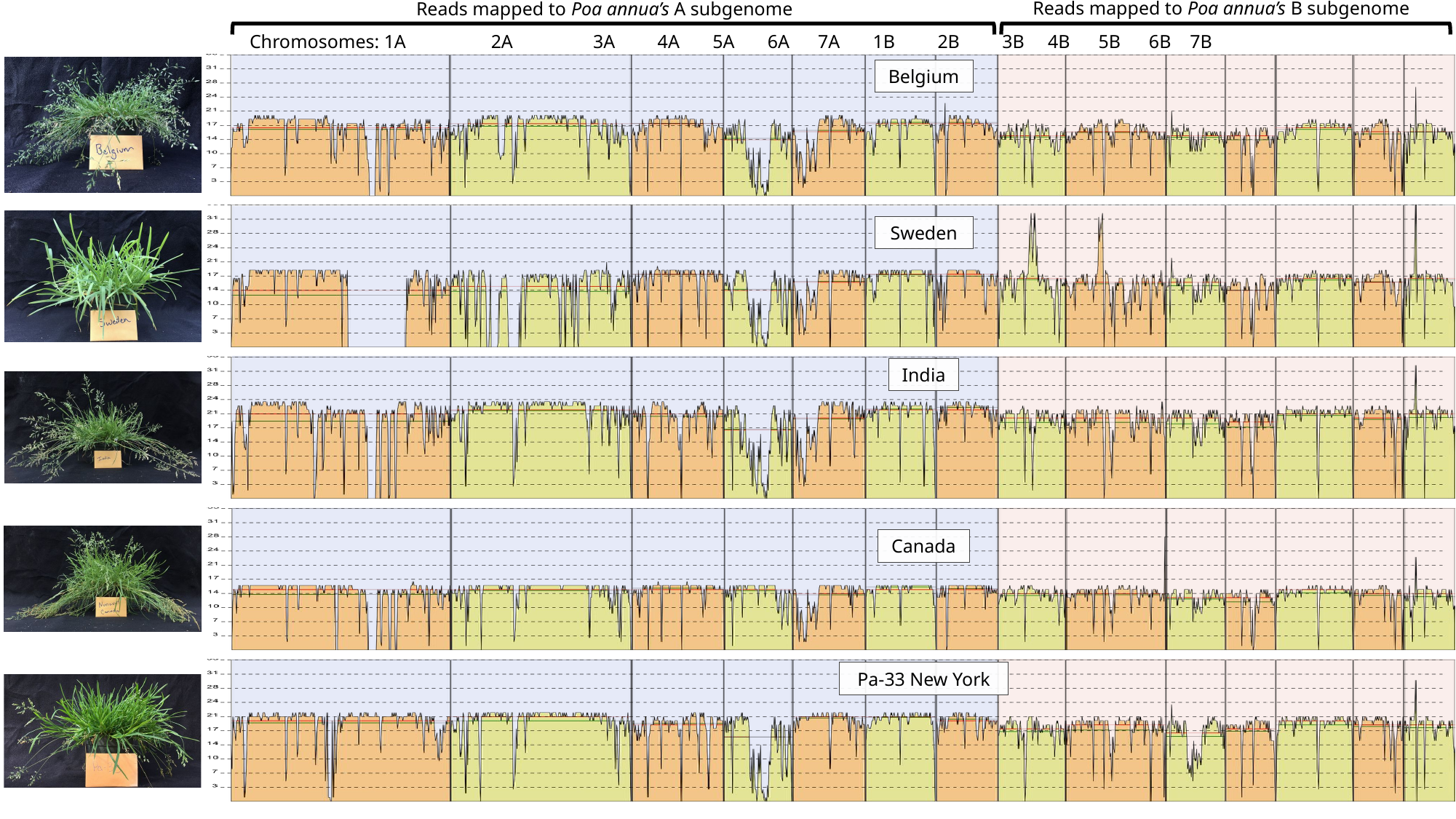

Reads mapped to Poa annua’s B subgenome
Reads mapped to Poa annua’s A subgenome
Chromosomes: 1A 2A 3A 4A 5A 6A 7A 1B 2B 3B 4B 5B 6B 7B
Supplementary Fig. 14 | Continued.
Belgium
Sweden
India
Canada
Pa-33 New York

## Slide 23
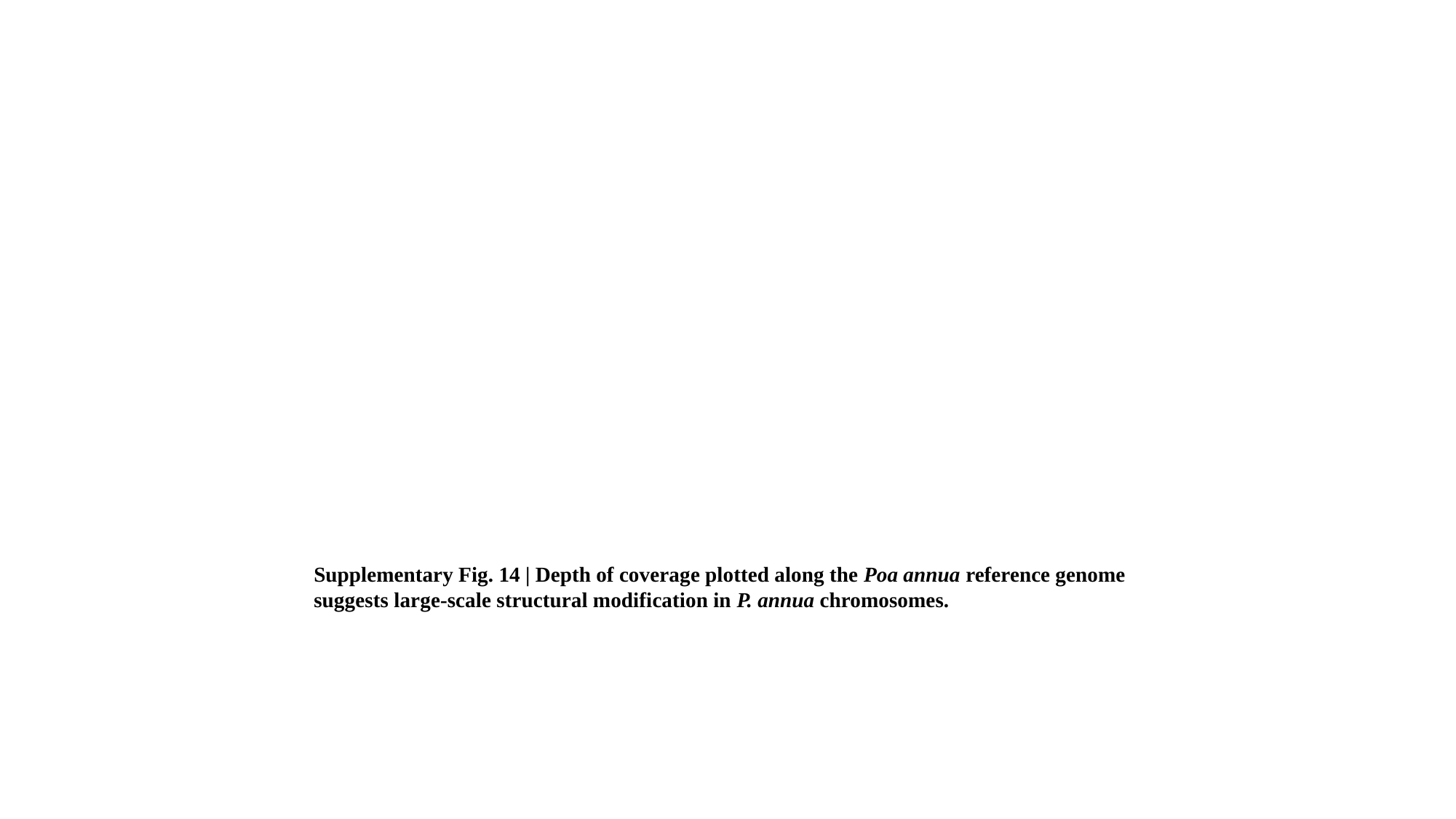

Supplementary Fig. 14 | Depth of coverage plotted along the Poa annua reference genome suggests large-scale structural modification in P. annua chromosomes.

## Slide 24
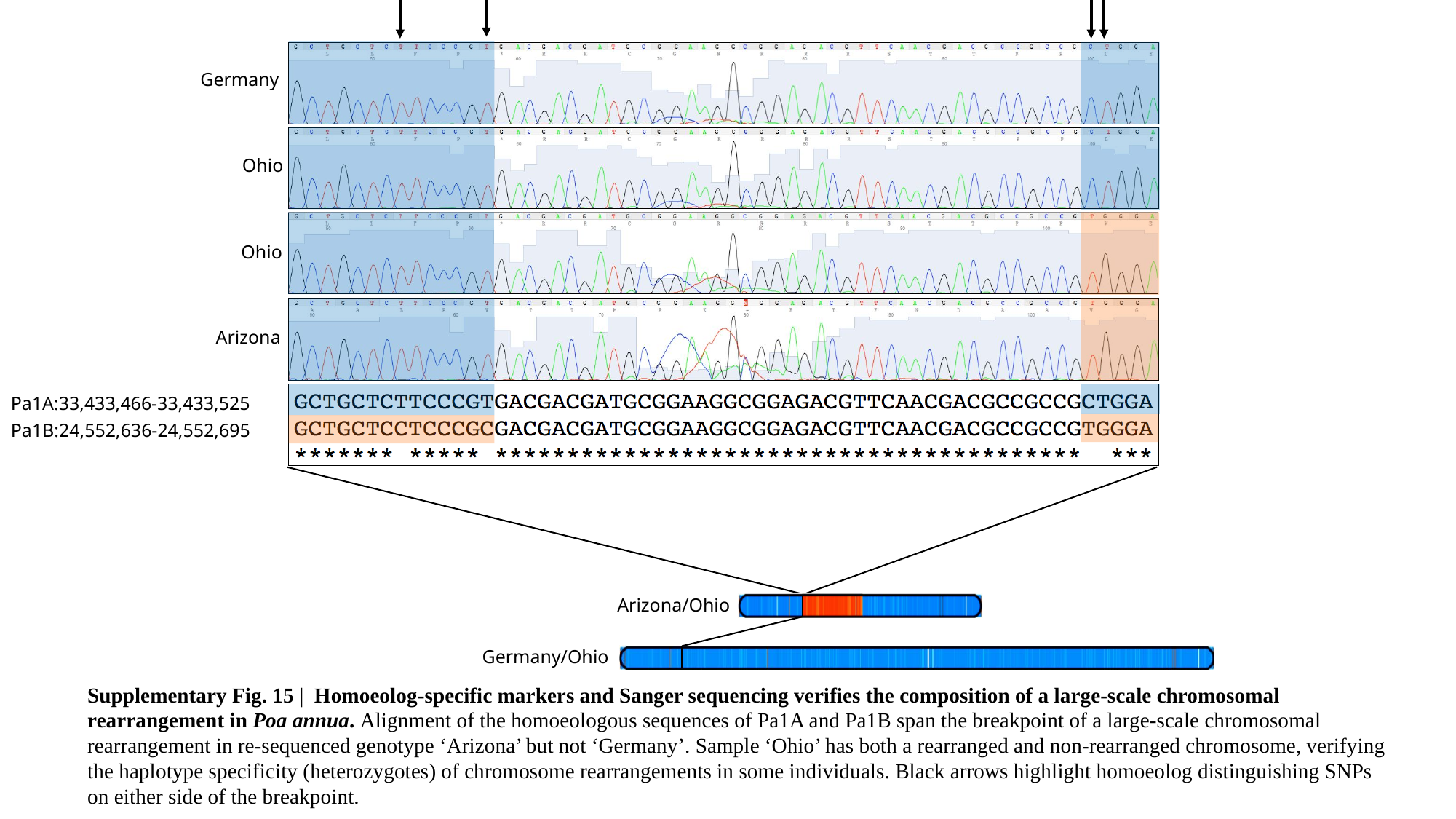

Germany
Ohio
Ohio
Arizona
Pa1A:33,433,466-33,433,525
Pa1B:24,552,636-24,552,695
Arizona/Ohio
Germany/Ohio
Supplementary Fig. 15 | Homoeolog-specific markers and Sanger sequencing verifies the composition of a large-scale chromosomal rearrangement in Poa annua. Alignment of the homoeologous sequences of Pa1A and Pa1B span the breakpoint of a large-scale chromosomal rearrangement in re-sequenced genotype ‘Arizona’ but not ‘Germany’. Sample ‘Ohio’ has both a rearranged and non-rearranged chromosome, verifying the haplotype specificity (heterozygotes) of chromosome rearrangements in some individuals. Black arrows highlight homoeolog distinguishing SNPs on either side of the breakpoint.

## Slide 25
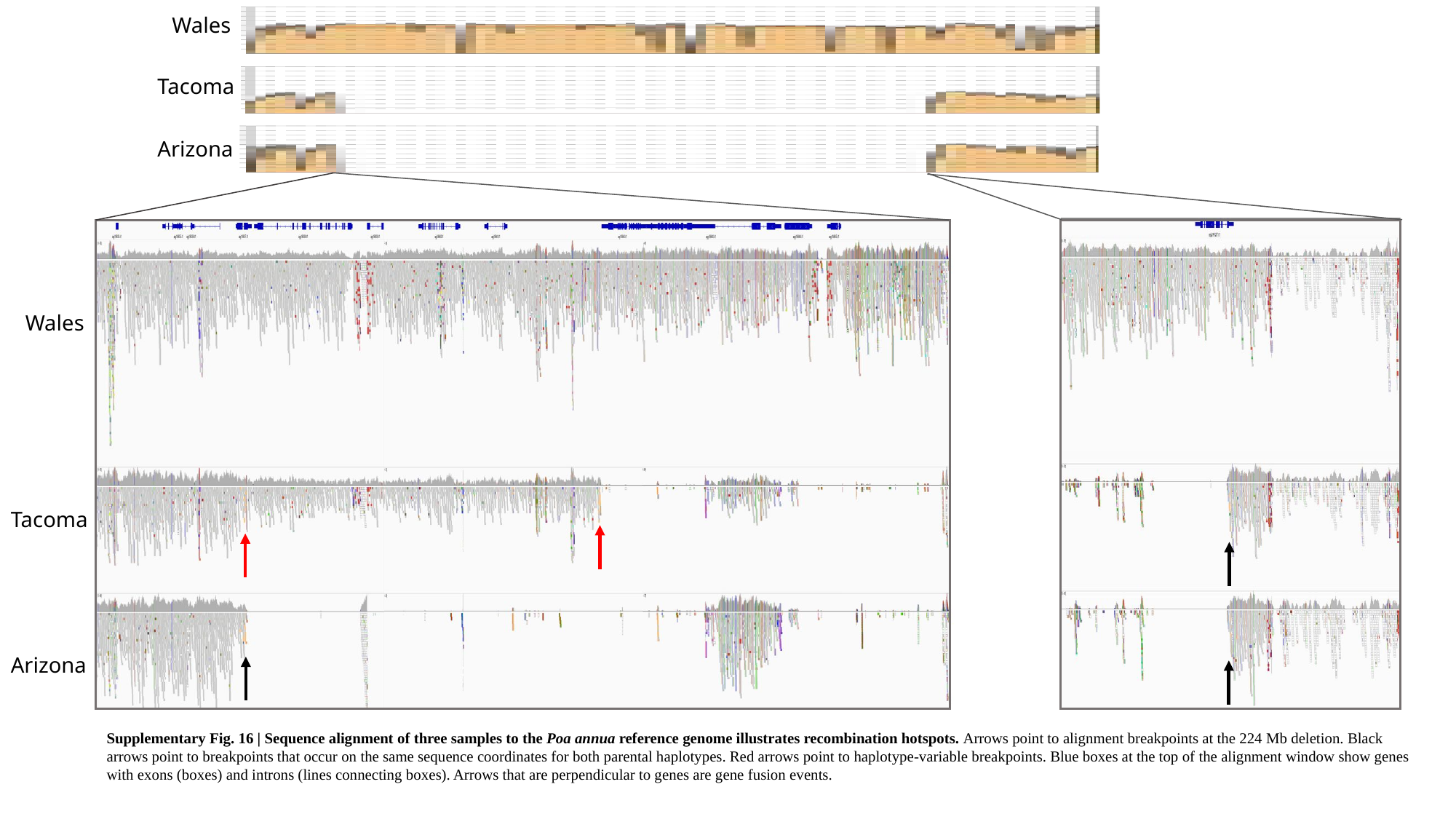

Wales
Tacoma
Arizona
Wales
Tacoma
Arizona
Supplementary Fig. 16 | Sequence alignment of three samples to the Poa annua reference genome illustrates recombination hotspots. Arrows point to alignment breakpoints at the 224 Mb deletion. Black arrows point to breakpoints that occur on the same sequence coordinates for both parental haplotypes. Red arrows point to haplotype-variable breakpoints. Blue boxes at the top of the alignment window show genes with exons (boxes) and introns (lines connecting boxes). Arrows that are perpendicular to genes are gene fusion events.

## Slide 26
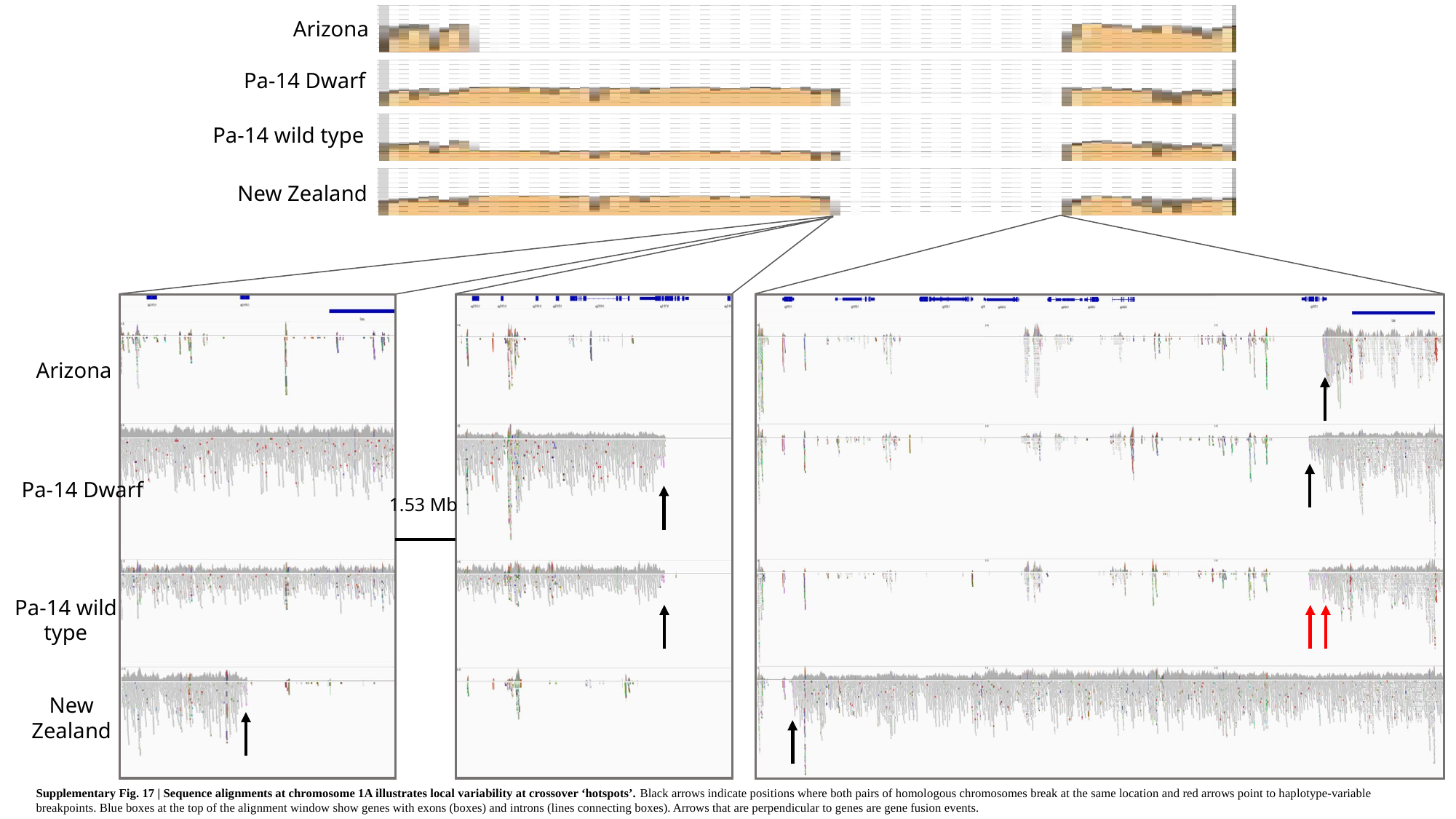

Arizona
Pa-14 Dwarf
Pa-14 wild type
New Zealand
Arizona
Pa-14 Dwarf
1.53 Mb
Pa-14 wild type
New Zealand
Supplementary Fig. 17 | Sequence alignments at chromosome 1A illustrates local variability at crossover ‘hotspots’. Black arrows indicate positions where both pairs of homologous chromosomes break at the same location and red arrows point to haplotype-variable breakpoints. Blue boxes at the top of the alignment window show genes with exons (boxes) and introns (lines connecting boxes). Arrows that are perpendicular to genes are gene fusion events.

## Slide 27
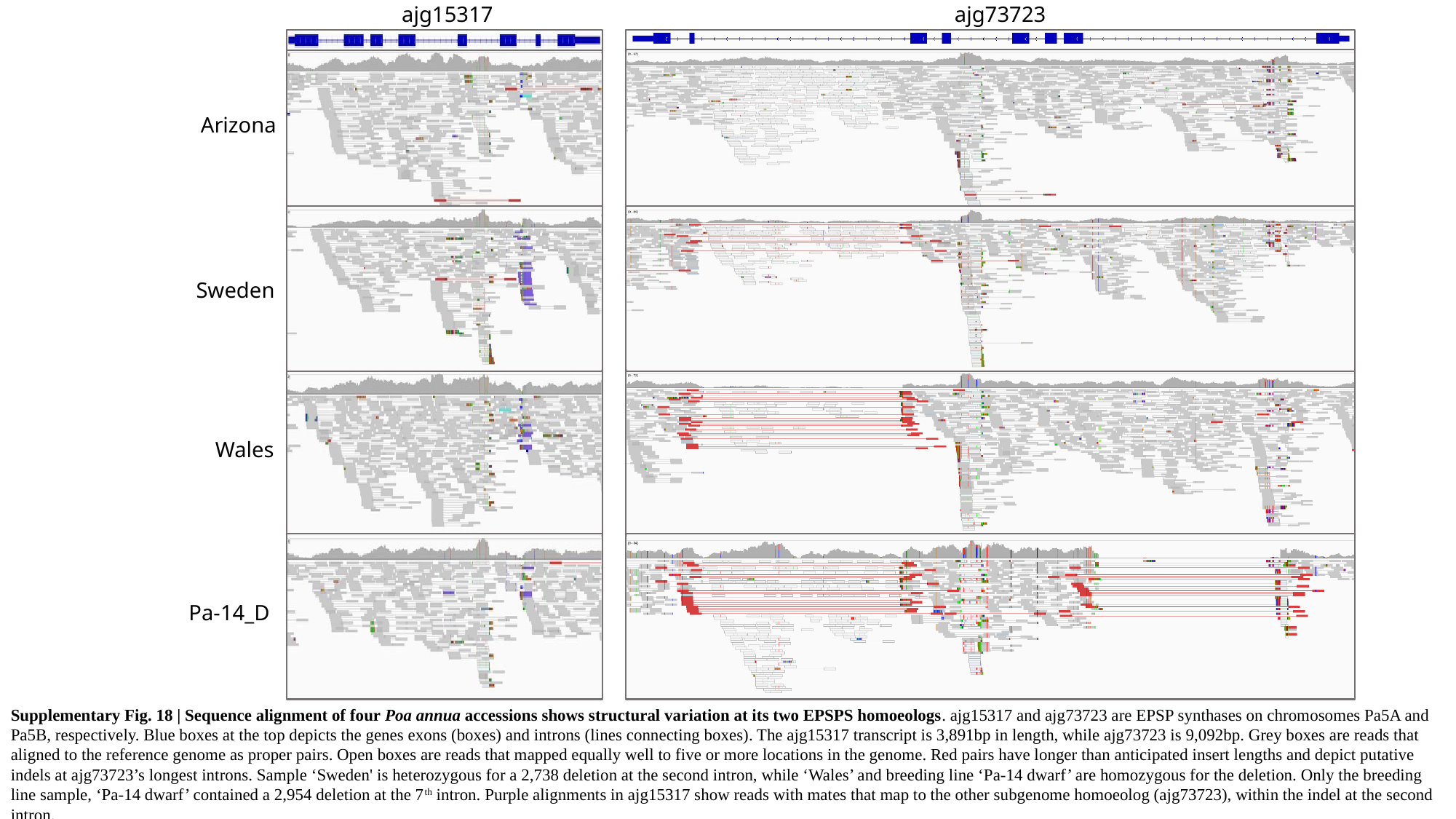

ajg15317
ajg73723
Arizona
Sweden
Wales
Pa-14_D
Supplementary Fig. 18 | Sequence alignment of four Poa annua accessions shows structural variation at its two EPSPS homoeologs. ajg15317 and ajg73723 are EPSP synthases on chromosomes Pa5A and Pa5B, respectively. Blue boxes at the top depicts the genes exons (boxes) and introns (lines connecting boxes). The ajg15317 transcript is 3,891bp in length, while ajg73723 is 9,092bp. Grey boxes are reads that aligned to the reference genome as proper pairs. Open boxes are reads that mapped equally well to five or more locations in the genome. Red pairs have longer than anticipated insert lengths and depict putative indels at ajg73723’s longest introns. Sample ‘Sweden' is heterozygous for a 2,738 deletion at the second intron, while ‘Wales’ and breeding line ‘Pa-14 dwarf’ are homozygous for the deletion. Only the breeding line sample, ‘Pa-14 dwarf’ contained a 2,954 deletion at the 7th intron. Purple alignments in ajg15317 show reads with mates that map to the other subgenome homoeolog (ajg73723), within the indel at the second intron.

## Slide 28
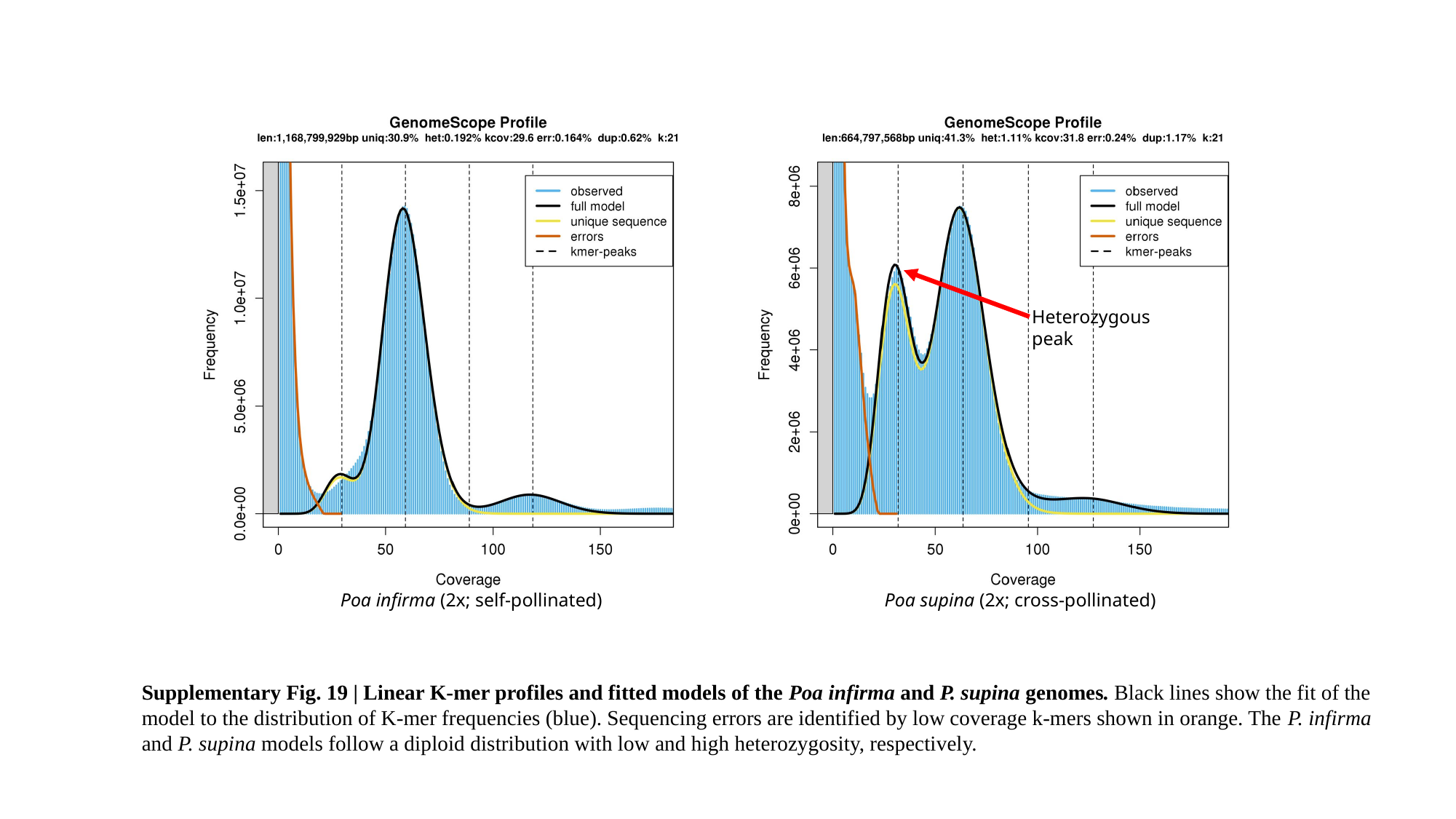

Heterozygous peak
Poa infirma (2x; self-pollinated)
Poa supina (2x; cross-pollinated)
Supplementary Fig. 19 | Linear K-mer profiles and fitted models of the Poa infirma and P. supina genomes. Black lines show the fit of the model to the distribution of K-mer frequencies (blue). Sequencing errors are identified by low coverage k-mers shown in orange. The P. infirma and P. supina models follow a diploid distribution with low and high heterozygosity, respectively.

## Slide 29
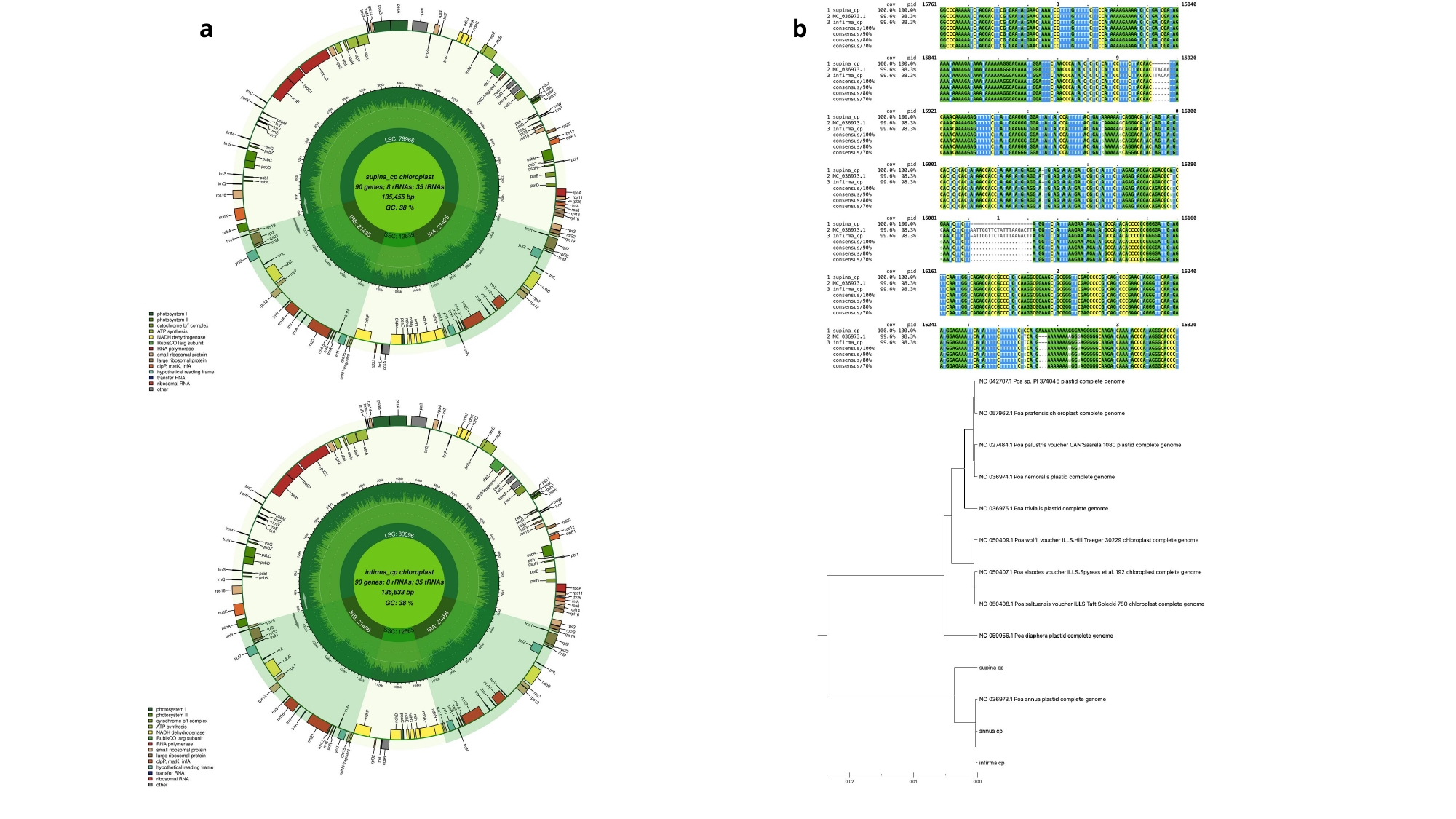

a
b
Supplementary Fig. 20 | The chloroplast sequences of Poa infirma and P. supina. (a) Chloroplast maps for P. supina and P. infirma. (b) Sequence alignment of chloroplasts show that P. annua’s maternal parent is P. infirma.
